# Risk factor-based screening compared to universal screening for gestational diabetes mellitus in marginalized Burman and Karen populations on the Thailand-Myanmar border: An observational cohort

**DOI:** 10.12688/wellcomeopenres.17743.1

**Published:** 2022-04-07

**Authors:** Janna T. Prüst, Tobias Brummaier, Mu Wah, Htay Htay Yee, Nyo Nyo Win, Mupawjay Pimanpanarak, Aung Myat Min, Mary Ellen Gilder, Nay Win Tun, Onaedo Ilozumba, Basirudeen Syed Ahamed Kabeer, Annalisa Terranegra, Francois Nosten, Sue J. Lee, Rose McGready

**Affiliations:** 1 Shoklo Malaria Research Unit, Mahidol–Oxford Tropical Medicine Research Unit, Faculty of Tropical Medicine, Mahidol University, Bangkok, 10400, Thailand; 2 Department of Health Sciences, Vrije Universiteit Amsterdam, Amsterdam, 1081, The Netherlands; 3 Swiss Tropical and Public Health Institute, Allschwill, 4123, Switzerland; 4 University of Basel, Basel, 4001, Switzerland; 5 Department of Family Medicine, Faculty of Medicine, Chiang Mai University, Chiang Mai, 50200, Thailand; 6 Research Department, Sidra Medicine, Doha, Qatar; 7 Centre for Tropical Medicine and Global Health, Nuffield Department of Medicine, University of Oxford, Oxford, OX3 7LG, UK; 8 Mahidol-Oxford Tropical Medicine Research Unit, Mahidol University, Bangkok, 10400, Thailand

**Keywords:** Gestational diabetes mellitus, HAPO trial, Maternal and neonatal anthropometry, Oral glucose tolerance test, Symphysis-fundal height measurements, Migrants, Risk-factor-based screening, thin-diabetic

## Abstract

**Background:** Gestational diabetes mellitus (GDM) contributes significantly to maternal and neonatal morbidity, but data from marginalized populations remains scarce. This study aims to compare risk-factor-based screening to universal testing for GDM among migrants along the Thailand-Myanmar border.

**Methods:** From the prospective cohort (September 2016, February 2019), 374 healthy pregnant women completed a 75g oral glucose tolerance test (OGTT) at 24-32 weeks gestation. Fasting, one hour and two hour cut-offs were based on Hyperglycaemia and Adverse Pregnancy Outcomes (HAPO trial) criteria and cases were treated. The sensitivity and specificity of risk-factor-based screening criteria was calculated using OGTT as the gold standard. Risk factors included at least one positive finding among 10 criteria, e.g., obesity (body mass index (BMI) ≥27.5kg/m ^2^), 1 ^st^ degree relative with diabetes etc. Adverse maternal and neonatal outcomes were compared by GDM status, and risk factors for GDM were explored.

**Results:** GDM prevalence was 13.4% (50/374) (95% CI: 10.3-17.2). Risk-factors alone correctly identified 74.0% (37/50) OGTT positive cases: sensitivity 74.0% (59.7-85.4) and specificity 27.8% (3.0-33.0). Burman women accounted for 29.1% of the cohort population, but 38.0% of GDM cases. Percentiles for birthweight (p=0.004), head circumference (p=0.005), and weight-length ratio (p=0.010) were higher in newborns of GDM mothers compared with non-GDM, yet 21.7% (75/346) of newborns in the cohort were small-for-gestational age. In Burman women, overweight/obese BMI was associated with a significantly increased adjusted odds ratio 5.03 (95% CI: 1.43-17.64) for GDM compared to normal weight, whereas underweight and overweight/obese in Karen women were both associated with similarly elevated adjusted odds, approximately 2.4-fold (non-significant) for GDM. GDM diagnosis by OGTT was highest prior to peak rainfall.

**Conclusions:** Risk-factor-based screening was not sufficiently sensitive or specific to be useful to diagnose GDM in this setting among a cohort of low-risk pregnant women. A two-step universal screening program has thus been implemented.

## Introduction

Gestational diabetes mellitus (GDM) is rising in tandem with obesity globally, including in South- and South-East Asia ^1^. Population characteristics such as urban or rural residence and the diagnostic method used, results in wide estimates such that in Thailand, for example, the GDM prevalence is estimated between 6.1% ^1^ and 29.2% ^2^. In Myanmar, there is insufficient data to provide reliable estimations of the prevalence ^1^. Detection of GDM is important as it is associated with neonatal macrosomia, neonatal hypoglycaemia and an increased risk for birth complications, such as shoulder dystocia and the need for caesarean section ^3– 5^. Furthermore, GDM is associated with an increased risk of preeclampsia, and entails a tenfold risk of developing type II diabetes ^6^ and doubles the risk of cardiovascular events later in life ^7^.

In absolute numbers, GDM predominantly affects women in low- and middle-income countries (LMIC), although at 13.5% relative estimates are similar in low-income countries compared to high-income countries ^8^ (HIC) at 13.4% ^9^. In HIC, migrant women have a higher risk for GDM and associated adverse birth outcomes ^10^. In South-East Asia, a large proportion of the population lives in rural areas with high poverty rates and lack of access to adequate health care ^11^. While most women do receive some form of antenatal care (ANC), screening for GDM is often not available ^12^. In addition, awareness of GDM is limited, as are adequate protocols and tools to monitor blood glucose, which hinders best-practice management ^12, 13^. In LMICs, antenatal care visits provide a small window for interventions to address the intergenerational cycle of malnutrition that is common in these settings ^10^.

In a meta-analysis, Lee *et al*. ^14^ described a GDM prevalence of 11.5% in Asian women and identified the following risk factors: multiparity, previous GDM, or pregnancy-induced hypertension (PIH), a family history of GDM and an increased maternal body mass index (BMI ≥25kg/m ^2^). An obstetric history of preterm birth, macrosomia, stillbirth, or an infant with congenital anomalies are also recognised GDM risk factors ^14^. In resource-limited settings, assessment of the uterus size by symphysis-fundal height measurement (SFH) as a proxy for foetal size has been suggested as a first level screening tool for foetal growth assessment. SFH measurement is a straightforward and inexpensive method, but its precision is controversial ^3^. A bespoke SFH growth curve was estimated for the pregnant Thailand-Myanmar border population ^15^; however, its applicability for GDM screening has not been assessed.

In 2020, the global migrant population was estimated to be 281 million, about 3.6% of the world’s total population ( World Migration Report 2020). According to the Thailand Ministry of Labour, there were 2,877,144 registered and an unknown number of undocumented migrants working in Thailand in 2019 ^16^. There are also an estimated 100,000 Karen and Burmese refugees in camps on the Thailand-Myanmar border. While the Shoklo Malaria Research Unit (SMRU) has provided health care to both the refugee and migrant population in its 30 plus year history, current efforts focus on humanitarian health care for migrants. In the pregnant migrant population attending SMRU ANC clinics, the nutrition transition has been marked by a two-fold increase in first trimester overweight measured by BMI in just over a decade ^17^, aggravated by limited awareness of healthy diets and lifestyle ^18^.

The environment has also been associated with GDM incidence, with positive associations with the warmer rather than the winter season, although this was less consistent when using actual measured temperature ^19^. A systematic review and meta-analyses evidenced 11 studies, all conducted in temperate countries, with no evidence from tropical countries ^19^. Nonetheless, undernutrition in the ‘hungry’ season, which coincides with the monsoon season when the previous year’s food crops become depleted before the current year’s harvest, has been associated with low birth weight and these infants grow up to have a higher risk of metabolic disease including diabetes ^20^.

Adequate GDM diagnosis and management improves maternal ^12^ and perinatal outcomes ^21^. Both universal and risk-factor-based screening are common practices, with no international consensus about best practice ^2, 22, 23^. Data remains scarce on GDM prevalence and its associated consequences in rural and marginalized populations. In 2011-2012, one of the first surveys conducted in a refugee camp reported a GDM prevalence of 10.1% (95% CI 6.2-14.0%) in Maela, the largest of the Thailand-Myanmar border camps ^22^. In this survey, GDM was significantly associated with increased maternal age and parity, and low literacy. Although the proportion of caesarean section and obesity were higher among women with GDM, this difference was not significant ^22^. In the low-resource setting of the refugee camp, the decision at that time was to commence efforts to screen for GDM based on risk factors using the Hyperglycaemia and Adverse Pregnancy Outcomes ^24^ criteria ^24^. SMRU implemented this approach in all its clinics on the border, i.e., for refugees and migrants. The applicability of the current GDM risk-factor-based screening policy in the migrant population has not been assessed.

This study aimed to evaluate the performance of the current risk-factor-based screening used in antenatal care clinics for migrant women to detect GDM compared to universal screening of all women. Within this cohort, adverse maternal and neonatal outcomes in women with and without GDM were evaluated and risk factors for GDM explored.

## Methods

### Ethical approval

The study was approved by the ethics committee of the Faculty of Tropical Medicine, Mahidol University, Bangkok, Thailand (Ethics Reference: TMEC 15–062, initial approval 1 December 2015), the Oxford Tropical Research Ethics Committee (Ethics Reference: OxTREC: 33–15, initial approval 16 December 2015) and reviewed by the local Tak Province Community Ethics Advisory Board. The study was conducted in full conformity with the Declaration of Helsinki and followed regulations of the ICH Guidelines for Good Clinical Practice.

### Study design

This study is reported in line with the STARD guidelines ^25^. Data was collected prospectively between September 2016 and February 2019 in women enrolled in their first trimester of pregnancy to an observational cohort study (ClinicalTrials.gov Identifier: NCT02797327) with GDM screening occurring from December 2016 to November 2018.

### Study setting

SMRU was established more than three decades ago and combines research and humanitarian work that serves the migrant population alongside the Thailand-Myanmar border. To be accessible within these communities, which largely depend on below minimum wage jobs, SMRU operates free-of-charge walk-in clinics offering universal antenatal care, as well as 24-hour delivery services, led by trained personnel originating from the local population.

At the same clinics, women may be invited to participate in research. The study was explained to all pregnant women attending SMRU ANC clinics in the first trimester and they were invited to participate if they met the study inclusion criteria and enrolled if consent was forthcoming. Informed consent was obtained in the form of a signature or in the event of an illiterate participant by thumbprint coupled with a confirmatory signature by an impartial literate witness.

### Sample size

A detailed description of the study protocol and SMRU routine ANC procedures are available elsewhere ^26^. Briefly, women were followed fortnightly throughout pregnancy, at delivery, and in the postpartum period. The planned sample size of 400 in the original study was based on estimated preterm birth rates (of approximately 8%) and on the following cohort inclusion criteria: a viable, singleton first trimester pregnancy and an unremarkable medical and obstetric history e.g., no history of caesarean section. For the secondary analysis of this cohort in relation to GDM risk-factor-based screening, additional exclusion criteria were miscarriage, maternal death, lost to follow-up, withdrawal of consent (primary cohort), and if OGTT was performed late (gestational age (GA) ≥33 weeks) or not done at all. Women who did not complete follow-up to delivery were replaced as permitted in the original protocol.

### Study variables

Baseline characteristics, regular prenatal check-ups for SFH, blood pressure, weight, and assessment of gestation by ultrasound, as well as birth outcomes, were collected by trained ANC staff and midwives in accordance with the study protocol. GA was estimated by crown rump length measured by first trimester ultrasound ^27^.

While the study protocol specified GDM screening with OGTT at 24–26 weeks of gestation, the Hyperglycaemia and Adverse Pregnancy Outcomes ^24^ study target time for testing was at 28 weeks (24–32 weeks). Therefore, OGTTs to 32 weeks of gestation were included in this study ^24^. In women with a history of GDM, an OGTT was performed as early as possible in pregnancy and repeated at 24–26 weeks if previously negative. GDM diagnosis was based on HAPO trial cut-offs: a fasting capillary blood glucose measurement of ≥92mg/dL, ≥180mg/dL one hour or ≥153mg/dL two hours after ingestion of 75g glucose were considered positive ^24^.

Since 2018, a fixed list of risk factors has been used to guide screening. These were derived by consensus from the refugee camp data ^24^ and local clinical experience e.g., including women with a history of difficult birth because GDM could result in an even more difficult birth in this pregnancy. Potential risk factors for GDM were collected at enrolment and throughout pregnancy. The local risk factor for GDM screening required at least one positive finding among the following 10 criteria: age ≥30, obesity (BMI ≥27.5kg/m ^2^), GDM in previous pregnancy, family history (1 ^st^ degree relative) of diabetes mellitus (although this is of reduced sensitivity in LMIC as access to diabetes screening is limited), previous macrosomia (≥4kg), previous caesarean section regardless of birth-weight, previous stillbirth, SFH ≥90th percentile, 2+/3+ glucose on a urine dipstick test, or polycystic ovarian syndrome (PCOS). As women with a previous caesarean section were excluded from the original study protocol, no PCOS were encountered, and there was no routine glucosuria screening, these criteria were not included in the analysis.

Serial symphysis-fundal height measurements (SFH) were included from 16 weeks of gestation on a two-weekly basis. After abdominal palpation, the SFH was measured from the pubic symphysis to the uterine fundus using a tape measure and rounded to the nearest centimetre ^28^. SFH data was examined using both local population centiles of 7,476 measurements in 2,467 women with an average height of 151cm ^15^ and international centiles based on 20,566 measurements in 4,239 women with an average height of 162cm ^29^.

Neonatal anthropometry (i.e., birthweight, head circumference, and length) were only considered if measured within 72 hours of birth. If women gave birth at SMRU, the neonate was weighed on a digital SECA 354 scale (precision 5g) with weekly calibration. Percentiles and z-scores for neonatal anthropometric parameters and for weight-length ratio (WLR) were calculated using standards as published by the Intergrowth-21 ^st^ Project ^30^. Born too small or large for GA (SGA, LGA) were defined as ≤10 ^th^ and ≥90 ^th^ percentile, respectively.

Gestational weight gain was defined as the final maternal weight measured not more than four weeks prior to birth, minus the weight measured at the first antenatal visit. For women with a normal BMI at enrolment (between 18.50 and 24.99kg/m ^2^), Intergrowth-21 ^st^ standard percentiles ^30, 31^ for each weight measurement from ≥26 weeks and ≤40 weeks of gestation were calculated.

### GDM management

If GDM was diagnosed, all women were counselled about lifestyle modification (e.g., diet and exercise), and their glucose levels were monitored weekly or every two weeks at the clinic. Treatment was provided either directly or if non-pharmacologic interventions led to insufficient glucose control, with metformin as the first choice and glibenclamide as an additional oral agent. Due to the lack of home-based glucose monitoring options and the absence of adequate storage facilities, insulin is only (and rarely) prescribed in this population when oral agents fail to provide adequate control.

### Statistical analysis

Data were analysed using SPSS, version 27 (IBM Corp. Armonk, NY, USA) (IBM SPSS Statistics, RRID:SCR_016479) and Stata, version 16.1 (TX, USA) (Stata, RRID:SCR_012763). Normally distributed continuous data were presented as means with standard deviation and non-normally distributed data as medians with interquartile range (IQR). Baseline characteristics as well as birth outcomes were compared between women with and without GDM. For continuous variables, the Student’s t-test or Mann-Whitney U test were used, and categorical variables were compared using the Fisher’s exact or Chi-square test. Univariate associations were quantified using logistic regression. To evaluate the predictive ability of the current screening approach to identify women with GDM, all risk factors were combined into one logistic regression model, using GDM as the outcome. The sensitivity and specificity of risk-factor-based screening criteria was calculated using OGTT as the gold standard. To identify risks and potential risk groups for GDM in this population, age (30 or older, vs. all others), smoking (yes/no), ethnicity (Karen and Burman), and BMI groups underweight, normal weight (reference group) and overweight/obese were explored using interaction terms and logistic regression modelling. Seasonality of GDM diagnosis was also explored by plotting the proportion of OGTT positive by month over the study period, against temperature and precipitation. Historic meteorological data was obtained from Weather Underground (weather station ID: IMAESO5), a service that provides real-time and historic weather information. Plots were created with the R package ggplot2 (RRID:SCR_014601) 

## Results

**Figure 1.**
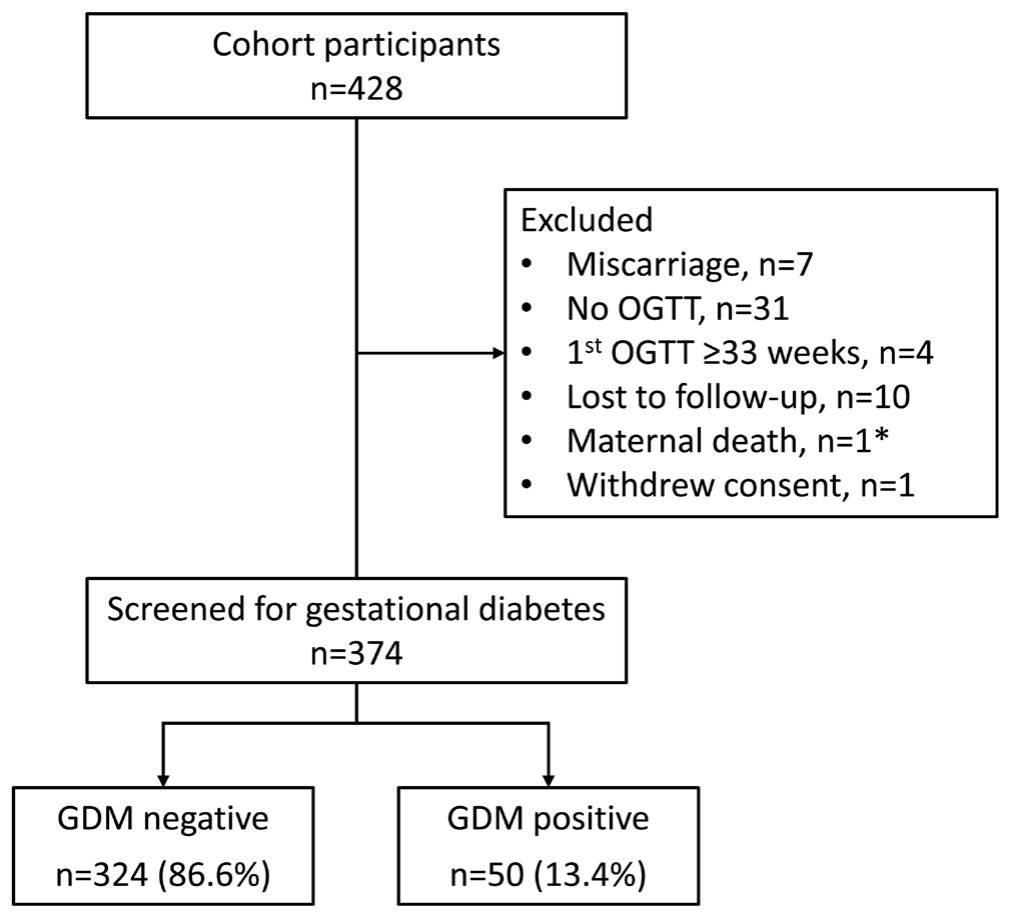
Flow diagram of participant selection. Abbreviations: GDM gestational diabetes mellitus, OGTT oral glucose tolerance test. * Sudden death due to mixed mitral valve disease at seven months gestation.

Following exclusions, 87.4% (374/428) of pregnant women from the original cohort were available for analysis ( Figure 1). Of these, 13.4% (50/374, 95% CI 10.3-17.2), were diagnosed with GDM by OGTT. The median (IQR) number of antenatal care visits was 16 (IQR 15–17). Baseline maternal characteristics of women with and without GDM were compared ( Table 1; ^32^). Women with GDM were significantly more likely to have had previous GDM (4.0% vs. 0, p<0.001), postpartum hypertension (4.0% vs. 0.3%, p=0.006) and non-significantly, to smoke (12.0% vs. 6.5%, p=0.161) and report prior macrosomia (2.0% vs. 0.31%, p=0.127). They were less likely to have had previous preterm labour (0% vs. 7.41%, p=0.047). A family history of diabetes was rarely reported (n=6) by women irrespective of GDM status (2.0% vs. 1.5%, p=0.811).

**Table 1.** Demographic characteristics of the cohort.

Characteristics	Total	Without GDM	With GDM	p-value
N	374	324	50	
Age (years), median [IQR]	25 [21, 30]	25 [21, 30]	24 [22, 28]	0.899
Age 30 and older, n (%)	99 (26.5%)	87 (26.9%)	12 (24.0%)	0.671
Ethnicity *, n (%)				0.333
Karen	247 (66.0%)	218 (67.3%)	29 (58.0%)	
Burman	109 (29.1%)	90 (27.8%)	19 (38.0%)	
Other	18 (4.8%)	16 (4.9%)	2 (4.0%)	
Gravidity, n (%)				0.935
Nulligravida	99 (26.5%)	86 (26.5%)	13 (26.0%)	
Multigravida	275 (73.5%)	238 (73.5%)	37 (74.0%)	
GA at enrolment (weeks), median [IQR]	9.6 [8.1, 11.6]	9.5 [8.0, 11.6]	9.9 [8.6, 11.7]	0.211
Literate, n (%)	240 (64.2%)	210 (64.8%)	30 (60.0%)	0.509
Smoking, n (%)	27 (7.2%)	21 (6.5%)	6 (12.0%)	0.161
BMI (kg/m ^2^), median [IQR]	20.6 [18.9, 23.3]	20.5 [19.0, 23.1]	21.0 [18.5, 24.4]	0.586
BMI 27.5kg/m ^2^ and higher, n (%)	23 (6.1%)	19 (5.9%)	4 (8.0%)	0.558
BMI <18.5kg/m ^2^, n (%)	73 (19.5%)	61 (18.8%)	12 (24.0%)	0.390
Height (cm) ^29^, mean ± SD	151.8 ± 4.8	151.7 ± 4.8	152.4 ± 4.7	0.369
MUAC (cm) ^29^, median [IQR]	25.9 [23.8, 28.3]	25.9 [23.9, 28.3]	25.4 [23.6, 28.9]	0.793
HIV, n (%)	0 (0.0%)	0 (0.0%)	0 (0.0%)	1.00
Syphilis, n (%)	6 (1.6%)	6 (1.9%)	0 (0.0%)	0.331
HepBsAg positive, n (%)	21 (5.6%)	17 (5.2%)	4 (8.0%)	0.431
Obstetric history, n (%)				
GDM	2 (0.5%)	0 (0.0%)	2 (4.0%)	<0.001
Vacuum delivery	3 (0.8%)	3 (0.9%)	0 (0.0%)	0.495
Macrosomia	2 (0.5%)	1 (0.3%)	1 (2.0%)	0.127
Stillbirth	6 (1.6%)	6 (1.9%)	0 (0.0%)	0.332
Miscarriage	93 (24.9%)	82 (25.3%)	11 (22.0%)	0.614
Previous preterm Labour	24 (6.4%)	24 (7.4%)	0 (0.0%)	0.047
Pregnancy Induced Hypertension	2 (0.5%)	2 (0.6%)	0 (0.0%)	0.577
Hypertension postpartum	3 (0.8%)	1 (0.3%)	2 (4.0%)	0.006
Family history of diabetes	6 (1.6%)	5 (1.5%)	1 (2.0%)	0.811

Abbreviations (alphabetic order): Ag antigen, BMI body mass index, GA gestational age, GDM gestational diabetes mellitus, IQR interquartile range, HepBsAg hepatitis B surface antigen, HIV human immunodeficiency virus, MUAC mid-upper arm circumference, SD standard deviation.

*Other includes Mon (n=8), Pa Oh (n=5), Rakhine (n=2), Shan (n=1), Ka Main (n=1), one patient self-identified as Muslim (n=1)

**Figure 2.**
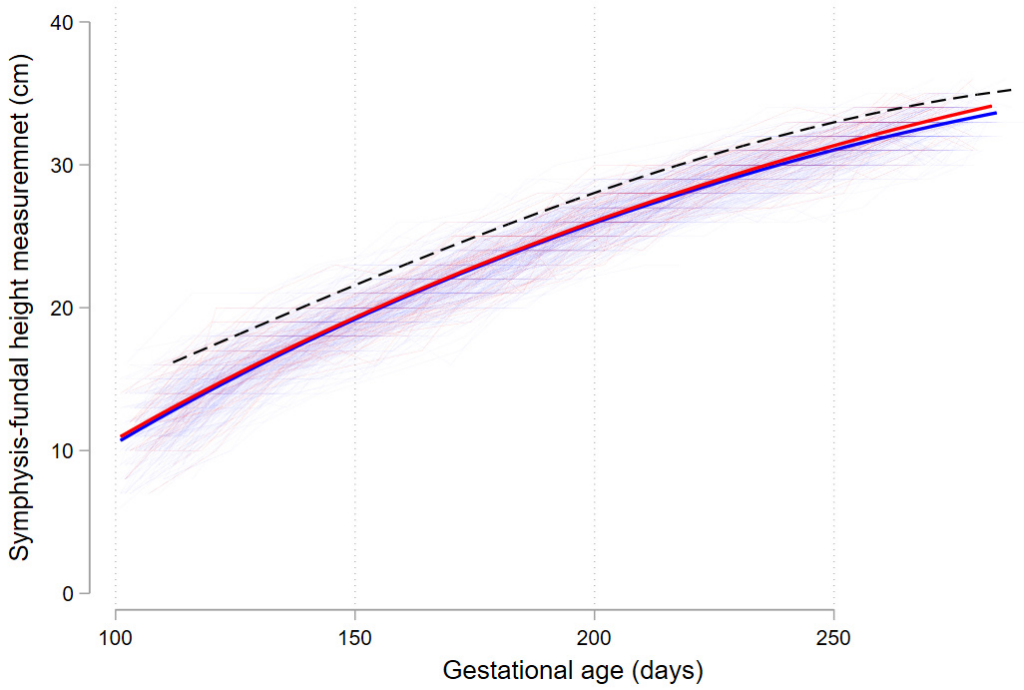
Symphysis-fundal height trajectories throughout pregnancy. Red lines indicate women with GDM (13.4%, n=50), blue lines women without GDM (86.6%, n=324). Dashed black line indicates the 90 ^th^ centile. Heavy red and blue lines represent fractional polynomial fit from individual measurements. Abbreviations: GDM gestational diabetes mellitus.

Overall, 23 women (6.1%) were obese (BMI ≥27.5kg/m ^2^) and this was similar in GDM positive compared to negative women (8.0% vs. 5.9%, p=0.558). Women who self-identified as being of Burman descent had a higher, albeit not statistically significant, GDM prevalence when compared with Karen and women of other ethnicities (17.4% (19/109) vs. 11.7% (29/247) and 11.1% (2/18), respectively, p=0.333). Burman women accounted for 29.1% of the cohort population, but 38.0% of GDM cases ( Table 1). Median gestational weight gain was similar between the two groups (p=0.982), but there were more women with GDM with an SFH ≥90 ^th^ centile during pregnancy with gestational week ≥24, 68.0% vs. 52.8%, p=0.044 ( Table 2). In particular, from about 224 days (32 weeks) onwards, women with GDM appeared to have larger SFH when compared with women without GDM ( Figure 2).

**Table 2.** Birth outcomes and neonatal anthropometry.

Birth outcomes and neonatal anthropometry	Total	Without GDM	With GDM	p-value
N	374	324	50	
GA at delivery (weeks), median [IQR]	39.6 [38.7, 40.1]	39.6 [38.8, 40.3]	39.1 [38.3, 39.9]	0.068
Gestational weight gain (kg), median [IQR]	10 [7, 12]	10 [7, 12]	10 [7, 12]	0.982
Weight gain ≥90 ^th^ centile	43/367 (11.7%)	38/319 (11.9%)	5/48 (10.4%)	0.764
SFH ≥90 ^th^ centile (GA ≥24), n (%)	205/374 (54.8%)	171/324 (52.8%)	34/50 (68.0%)	0.044
Preterm birth, n (%)	18/374 (4.8%)	17/324 (5.2%)	1/50 (2.0%)	0.318
Stillbirth, n (%)	4/374 (1.1%)	4/324 (1.2%)	0/50 (0.0%)	1.000
Mode of delivery				
Vaginal delivery, n (%)	352/374 (94.1%)	304/324 (93.8%)	48/50 (96.0%)	0.543
Caesarean Section, n (%)	20/374 (5.3%)	18/324 (5.6%)	2/50 (4.0%)	0.649
Place of labour				0.905
SMRU clinic, n (%)	301/374 (80.5%)	259/324 (79.9%)	42/50 (84.0%)	
Home, n (%)	27/374 (7.2%)	25/324 (7.7%)	2/50 (4.0%)	
Hospital, n (%)	37/374 (9.9%)	32/324 (9.9%)	5/50 (10.0%)	
Other, n (%)	9/374 (2.4%)	8/324 (2.5%)	1/50 (2.0%)	
Induction of labour, n (%)	25/373 (6.7%)	22/323 (6.8%)	3/50 (6.0%)	0.831
Augmentation of labour, n (%)	36/373 (9.7%)	31/323 (9.6%)	5/50 (10.0%)	0.929
Length of ROM (min), median [IQR]	36 [5, 160]	35 (5, 156)	65 (7, 217)	0.287
Postpartum haemorrhage ‡, n(%)	19/352 (5.4%)	18/304 (5.9%)	1/48 (2.1%)	0.274
Perineum				0.604
Intact, n (%)	160/303 (52.8%)	136/261 (52.1%)	24/42 (57.1%)	
1 ^st^ or 2 ^nd^ degree tear, n (%)	134/303 (44.2%)	116/261 (44.4%)	18/42 (42.9%)	
Episiotomy, n (%)	9/303 (3.0%)	9/261 (3.4%)	0/42 (0.0%)	
Infant sex (male), n (%)	181/373(48.5%)	155/323 (48.0%)	26/50 (52.0%)	0.597
Median Apgar score [IQR] at one min	9 [9, 9]	9 [9, 9]	9 [9, 9]	0.825
Median Apgar score [IQR] at five min	10 [10, 10]	10 [10, 10]	10 [10, 10]	0.620
Neonatal resuscitation, n (%)	8/361 (2.2%)	8/313 (2.6%)	0/48 (0.0%)	0.263
Abnormal newborn exam, n (%)	4/373 (1.1%)	4/323 (1.2%)	0/50 (0.0%)	1.00
Infant weight (g), mean ± SD	2972 ± 402	2952 ± 398	3096 ± 408	0.019
Large for GA (>p90), n (%)	7/346 (2.0%)	4/297 (1.3%)	3/49 (6.1%)	0.028
Small for GA (<P10), n (%)	75/346 (21.7%)	68/297 (22.9%)	7/49 (14.3%)	0.175
Percentile *, median [IQR]	24.8 [11.6, 47.6]	23.2 [11.2, 43.9]	40.5 [16.3, 61.0]	0.004
Head circumference ^28^, mean ± SD	32.8 ± 1.3	32.7 ± 1.3	33.3 ± 1.3	0.005
Length ^28^, mean ± SD	48.2 ± 2.0	48.1 ± 2.0	48.4 ± 1.8	0.358
Weight-length ratio (%), mean ± SD	6.2 ± 0.7	6.1 ± 0.7	6.4 ± 0.7	0.010

Abbreviations (alphabetic order): GA gestational age, GDM gestational diabetes mellitus, IQR interquartile range, min minutes, ROM rupture of membranes, SD standard deviation, SFH symphysis fundal height, SMRU Shoklo Malaria Research Unit.

*birth weight for GA and sex, ‡ >500ml blood loss

### Birth outcomes

Newborns from mothers with GDM were heavier (mean birthweight (SD): 3096g (408) vs. 2952g (398), p=0.019), and nearly five times more likely to be large for GA (OR 4.78, 95% CI 1.04-22.1). They were also more likely to be in a higher percentile for birthweight adjusted for GA and sex (median (IQR): 40.5 (16.3, 61.0) vs. 23.2 (11.2, 43.9), p=0.004), have a larger head circumference (mean (SD): 33.3cm (1.3) vs. 32.7cm (1.3), p=0.005) and to have a higher weight-length ratio (mean (SD): 6.4% WLR (0.7) vs. 6.1% w/l (0.7), p=0.010), Table 2. Overall, the proportion of SGA was relatively high (21.7%, 75/346). Other adverse birth outcomes such as stillbirth (0%, 0/50 of GDM positive; 1.2%, 4/324 of GDM negative), and preterm birth (2.0%, 1/50 in GDM positive; 5.2%, 17/324 of GDM negative) were low.

### Risk-factor-based screening for GDM

Of the women with GDM, 88.0% (44/50) had only one of the three glucose measurements above the cut-offs. Testing as practiced in some settings to reduce costs, with fasting and two-hour tests would result in only 66% (33/50) of the GDM cases being detected ( Table 3). Of the 50 OGTT positive cases, 37 were correctly identified by risk factors alone, resulting in a sensitivity of 74.0% (59.7%-85.4%). Specificity was low, with 90 of 324 being correctly identified as negative for GDM using risk-factor-based screening: 27.8% (23.0%-33.0%). The positive and negative predictive values were 13.7% (9.8%-18.3%) and 87.4% (79.4%-93.1%), respectively.

**Table 3.** Details of OGTT test result and GDM treatment.

OGTT test results and GDM treatment	Total	Without GDM	With GDM	p-value
N	374	324	50	
GA (weeks) at OGTT, median [IQR]	26.6 [25.7, 27.6]	26.6 [25.7, 27.6]	26.6 [25.9, 27.4]	0.949
				
OGTT * results (mg/dL), median [IQR]				
BSL fasting	79 [74, 84]	78 [73, 83]	86 [81, 96]	<0.001
BSL one hour	132 [114, 154]	129 [112, 147]	173 [142, 191]	<0.001
BSL two hours	111 [97, 127]	110 [96, 123]	129 [113, 157]	<0.001
Proportion positive at each timepoint				
Fasting only			17 (34%)	
One hour only			17 (34%)	
Two hours only			10 (20%)	
Fasting and one hour			2 (4%)	
Fasting and two hours			0 (0%)	
One hour and two hours			3 (6%)	
All three			1 (2%)	
GDM treatment, n (%)				
Diet and exercise only			18 (36%)	
Diet & metformin			27 (54%)	
Metformin and glibenclamide			4 (8%)	
Metformin and insulin (uncontrolled on oral)			1 (2%)	

Abbreviations (alphabetic order): BSL blood sugar level, GA gestational age, GDM gestational diabetes mellitus, HAPO Hyperglycaemia and Adverse Pregnancy Outcomes, IQR interquartile range, OGTT oral glucose tolerance test.

*HAPO cut points in GDM: fasting, one hour and two hours BSL are ≥92, ≥180 and ≥153mg/dL, respectively.

Of the seven risk-factor-based screening items included in this analysis, a history of GDM and previous stillbirth could not be included in a multivariable model due to zero counts. None of the risk-factor-based screening criteria significantly predicted GDM status in this migrant population. History of macrosomia had a positive (wide confidence interval) and non-significant association due to the small number of cases (6.59, 95% CI 0.41-107.1, p=0.185). All other risk factors were not significant at p>0.20.

### GDM management and treatment

Approximately two out of three women, 64% (32/50), were medicated for their GDM ( Table 3). Most received metformin only (54% (27/50)), with a smaller proportion receiving metformin plus glibenclamide (8.0% (4/50)), and only one patient (2.0%) received insulin due to metformin failure at 27+3 weeks of gestation. This case required referral to the government hospital.

### GDM risk in Burman and Karen ethnic groups

Risk factors for GDM were examined separately for the two main ethnic groups in the population by multivariate analysis ( Table 4). After adjustment, overweight or obese Burman women were at a five-fold higher risk of GDM. A different relationship between BMI and GDM was apparent for Karen women where the risks were similarly elevated (non-significant) for both underweight and overweight or obese women ( Table 4).

**Table 4.** Risk factors for GDM in Karen and Burman women.

Risk factors	Karen n=247				Burman n=109			
	No GDM, n=218	GDM, n=29	Adjusted Odd Ratio (95% CI)	p-value	No GDM, n=90	GDM, n=19	Adjusted Odd Ratio (95% CI)	P-value
Age 30 and older, n (%)	56 (25.7)	6 (20.7)	0.52 (0.18-1.52)	0.231	24 (26.7)	5 (26.3)	0.54 (0.15-1.92)	0.343
Smoker, n (%)	19 (8.72)	5 (17.2)	3.09 (0.92-10.39)	0.069	2 (2.22)	1 (5.26)	5.27 (0.39-71.88)	0.213
BMI, kg/m ^2^								
Normal (18.50-22.99)	126 (57.8)	11 (38.0)	reference		46 (51.1)	6 (31.6)	reference	
Underweight (≤18.5)	31 (14.2)	7 (24.1)	2.41 (0.85-6.79)	0.097	26 (28.9)	4 (21.1)	1.20 (0.30-4.73)	0.704
Overweight / obese (≥23)	61 (28.0)	11 (37.9)	2.36 (0.95-5.89)	0.064	18 (20.0)	9 (47.4)	**5.03 (1.43-17.64)**	**0.012**

Data are shown in n (%) unless otherwise indicated. Abbreviations (alphabetic order): BMI body mass index, GDM gestational diabetes mellitus.

### GDM and seasonality

Seasonality of the proportion of women diagnosed each month was plotted against the total monthly rainfall ( Figure 3a) and the average monthly temperature ( Figure 3b). Although the numbers were small, peaks of GDM diagnosis appeared consistently raised in June: in 2017, of 17 screened women, five were GDM positive (29.4%) and five of nine in 2018 (55.6%). Monthly mean temperature demonstrated minor variation ranging from 24.1 °C to 29.7 °C. The two highest mean temperatures occurred in March, two months before the peaks in GDM diagnosis ( Figure 3b). There was a positive association in GDM diagnosis and monthly rainfall, which peaked in July in both years of the study period (p=0.053).

**Figure 3a.**
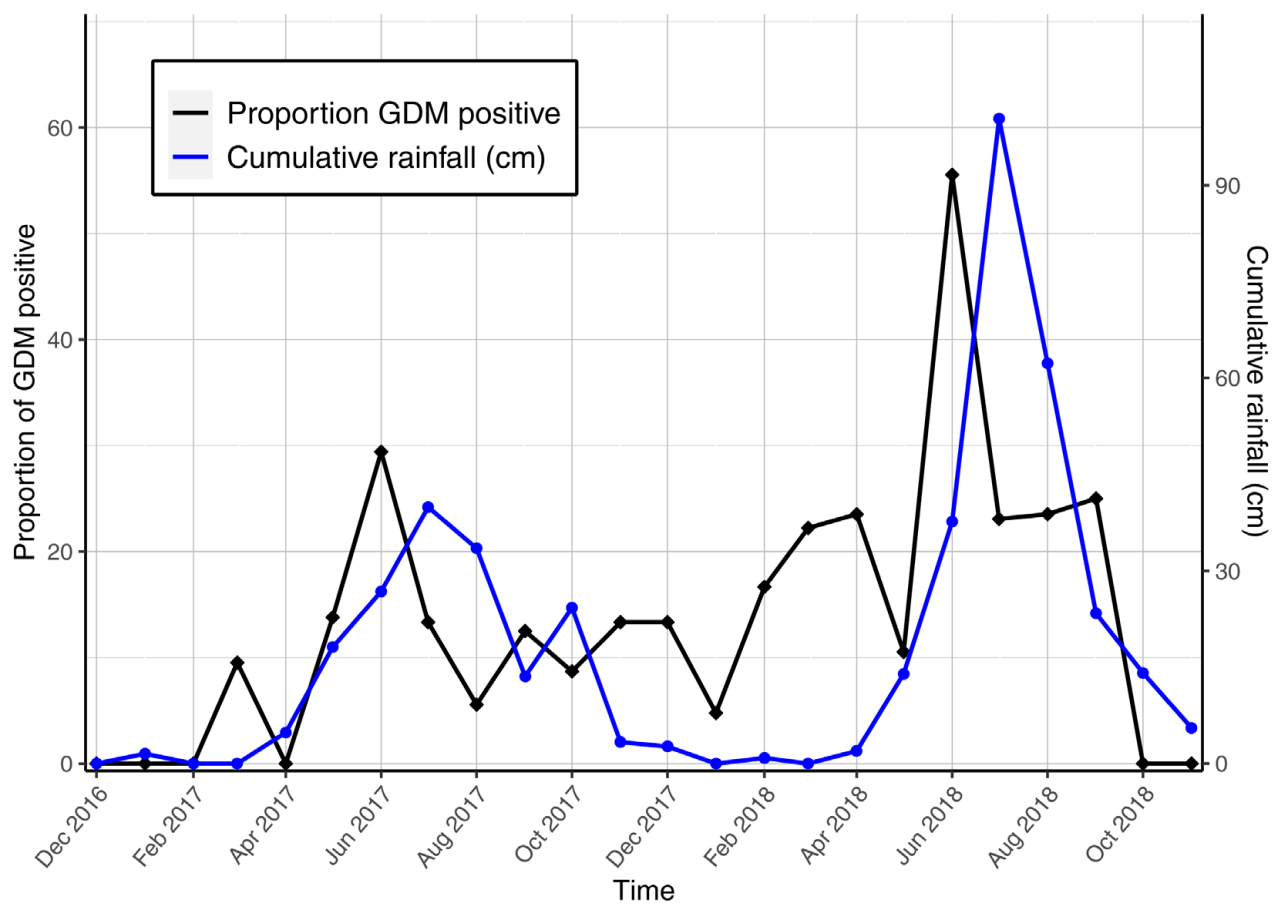
Seasonality of GDM diagnosis with cumulative monthly rainfall. Abbreviations: GDM gestational diabetes mellitus.

**Figure 3b.**
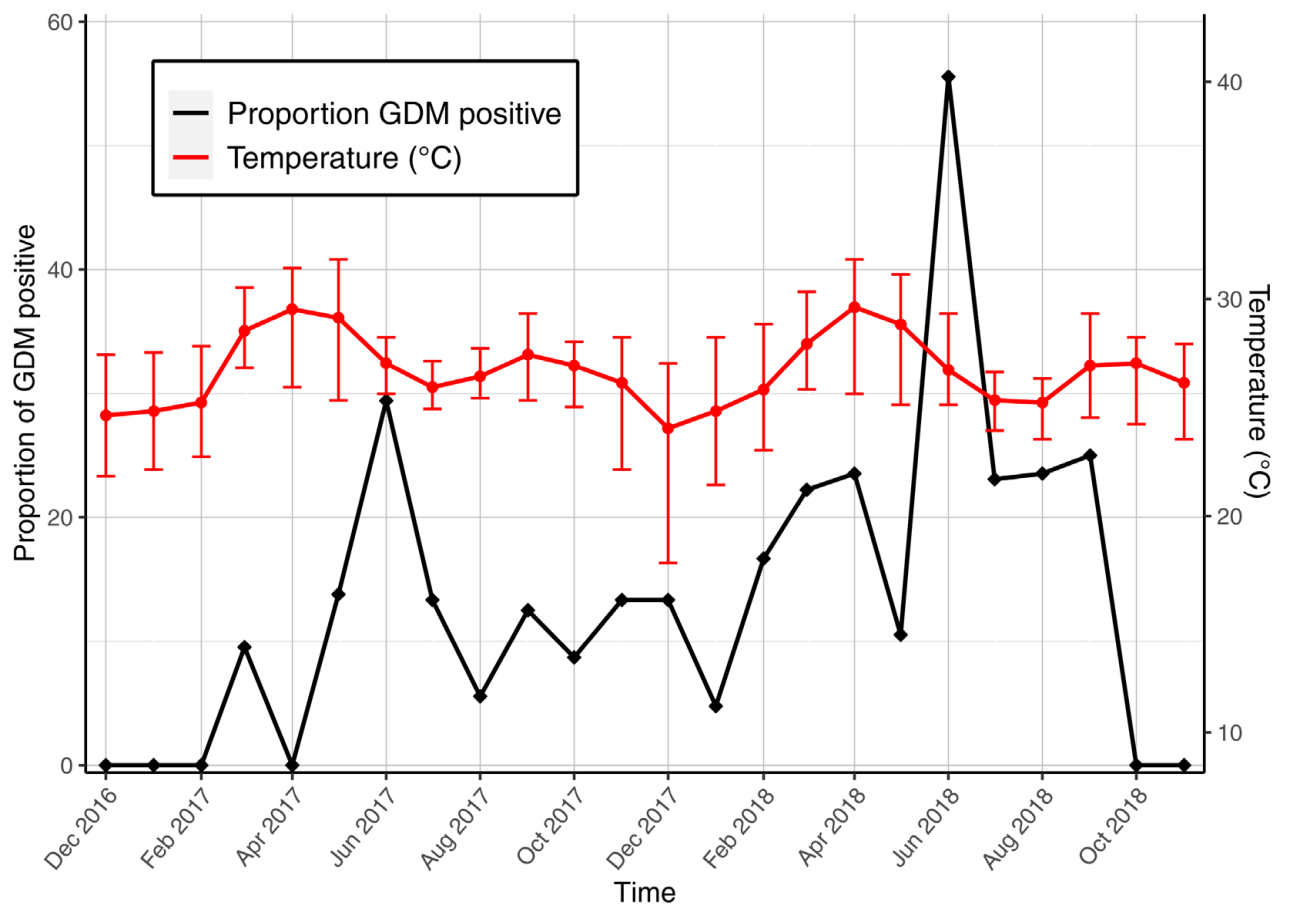
Seasonality of GDM diagnosis with average monthly temperature (deg. C). The error bars indicate maximum and minimum temperatures. Abbreviations: GDM gestational diabetes mellitus.

## Discussion

At least one in seven ‘healthy’ migrant women presenting to antenatal care in this study cohort had GDM based on the 75g OGTT. This analysis identified the shortcomings of current clinical practice as almost one in four women with GDM would have been missed based on risk-factor-based selection for screening. While the risk-factor-based screening had sensitivity 74.0% (95% CI 59.7-85.4), it lacked specificity 27.8% (95% CI 23.0-33.0) and resulted in a grossly inadequate positive predictive value of 13.7% (95% CI 9.8-18.3). Reasons for this poor performance could be related to the limited size of the cohort; due to the ‘healthy woman’ criteria and exclusion of those with a previous caesarean section (potentially due to undiagnosed GDM) from the original cohort; or that risk-factor-based screening is inherently weak for GDM diagnosis in Asian women. The low incidence of reported prior history of GDM or family history of diabetes, most likely results from the limited extent of testing in this population that has limited access to health care ^33^. The SFH >90 ^th^ centile, which improves the sensitivity of the risk-factor-based screening, is detectable from 32 weeks onwards, which is late in gestation to initiate treatment for GDM.

This evaluation identifies GDM as a significant health problem in Burman and Karen migrants on the Thailand-Myanmar border, similar to other migrant populations globally who have to make food choices based on limited expenditure ^34^. The BMI-related differences in risk factors observed on regression analysis for GDM in Karen and Burman women may relate to different diets and smoking habits between these ethnic groups. A more detailed dietary analysis based on quantitative 24-hour food recall is currently under evaluation. The similar odds for GDM in underweight and overweight/obese Karen women may be related to the thin-type II diabetic phenotype where individuals are at increased risk at a lower BMI ^35^. Gujral *et al*. ^36^ and Rajakramikan *et al.* ^37^ have proposed pathogenic mechanisms including: impaired insulin secretion, *in utero* undernutrition, or epigenetic alterations in the genome, to explain thin-type II diabetes. Of greatest concern is the propensity for this group of patients with undernutrition to have worse diabetes.

One of the novel findings of the study is the association between GDM just prior to the peak of the rainy season. Whether GDM is related to food type and availability at this period or a result of epigenetic changes, e.g., if these mothers were born during the hungry season and are pre-programmed to respond to nutrition differently, which increases the risk of metabolic diseases as adults, including in pregnancy, as hypothesized in the ‘Developmental Origins of Health and Disease’, is unknown ^20^.

Published studies in high-income settings have demonstrated a significant increase in perinatal morbidity in women with uncontrolled GDM compared to women with adequately treated GDM ^38^. In this analysis, there was a positive association between GDM and higher percentiles for infant birthweight, head circumference and weight-length ratio composition ^39– 41^ but no difference was seen in mode of delivery, postpartum haemorrhage, perineal damage or Apgar score by GDM status. Given that pregnant women with an unremarkable medical and obstetric history were prioritized in the cohort and women with GDM received treatment following the abnormal OGTT result, the low rate of adverse birth outcomes is not unexpected. The high rate of small for GA (one in five) newborns has been reported previously and highlights the double burden of nutrition in this population ^17^ but may also signal a risk for thin-type II diabetes ^35^.

Early detection of GDM may prevent the need for caesarean section, which limits total expenditure per pregnancy. While the cost for an individual OGTT is small (i.e., approximately 18 THB (0.54 USD) for one glucose test strip, 7.5 THB (0.22 USD) for 75g glucose powder), costs add up if thousands of pregnant women are universally screened each year. Considering the average cost for caesarean section in 2020 for migrant women was 27,695 THB (approximately 824 USD) referred to the public hospital system, one averted caesarean section would be equivalent to 1,539 glucose test strips – enough for OGTT in 500 women. Mo *et al*. ^42^ concluded that cost effectiveness of universal GDM screening is likely favourable over screening of targeted high-risk populations in a meta-analysis in mostly HIC, while others suggest that universal screening is not useful ^43^. Since access to adequate diabetes monitoring and pharmacological intervention is severely limited outside of pregnancy in resource-limited settings, there may be added benefit to universal screening in LMIC. The counselling women receive during pregnancy about their GDM in LMIC may be the first and only information provided on lifestyle modification to prevent the development of type II diabetes later in life ^44^. Reducing from three (fasting, one hour, two hours) to two (fasting, two hours) tests to reduce costs is not a useful alternative in this population as nearly nine in 10 were positive at a single timepoint distributed across all three time points. As the majority (68.7%) of GDM positive women in this study used pharmacological hypoglycaemic agents, there is a need for a better understanding of effective lifestyle interventions in this marginalized group ^2, 18, 45^.

The data on SFH contributes to the ongoing debate on the use of population-based vs. local centiles. As well as the significantly higher proportion of women with SFH ≥90 ^th^ centile from 24 weeks with GDM compared to women without GDM, there were 54.8% of all women with at least one SFH >90 ^th^ centile from 24 weeks, making this a useful and affordable tool. The proportion ≥90 ^th^ centile using local vs. the few SFH measurements that fall above the SFH 90 ^th^ centile using international centiles differs markedly. Using international standards, most GDM positive women would not be signalled as women with a problem in this population ^29^. This most likely arises from the greater than 10cm difference in maternal height between the populations participating in the cohorts for the centile curves. Both SFH centile methods have merits, but their limitations need to be understood by obstetric practitioners.

### Strengths of this study

The strengths of this study include first trimester enrolment and ultrasound dating allowing accurate assessment of neonatal anthropometry based on gestation. The risk of information bias is reduced by the prospective cohort design with minimal missing data. There was also close monitoring throughout pregnancy with a high number of antenatal care visits (median 16, IQR 15–17). Furthermore, weight and SFH were measured with calibrated instruments and by well-trained personnel. In addition, this analysis has had a direct local impact resulting in the implementation of universal screening with a two-step approach.

### Potential study limitations

Women with a complicated obstetric or medical history were excluded from the original study. As SMRU does not perform caesarean section in their own clinic, women thought to be at risk of this pregnancy complication were excluded from the original study as they were predicted to not be able to provide a complete set of samples. This was a selection bias for healthier pregnant women, potentially leading to an underestimate of the GDM prevalence in this border population, i.e., the study likely presents the minimum GDM rate in the community of pregnant women.

## Conclusions

These findings imply that GDM is a problem, more so in Burman than Karen migrants at the Thailand-Myanmar border, with overweight/obese Burman women at the highest risk. GDM determined by risk-factor-based screening performed poorly in this rural, resource-constrained pregnant population. Access to universal screening for GDM can potentially reduce negative impacts for an individual pregnancy but also provide an opportunity to reduce the onset of type II diabetes in marginalized populations undergoing rapid nutrition transition.

## Data availability

### Underlying data

Oxford University Research Archives: MSP COHORT GDM SCREEN. https://doi.org/10.5287/bodleian:j1vV56VJq ^32^

Data are available under the terms of the Creative Commons Attribution 4.0 International license (CC-BY 4.0).

### Reporting guidelines

**Figshare: STARD checklist for ‘**Risk factor-based screening compared to universal screening for gestational diabetes mellitus in marginalized Burman and Karen populations on the Thailand-Myanmar border: an observational cohort’. https://doi.org/10.6084/m9.figshare.19382624 ^25^

Data are available under the terms of the Creative Commons Attribution 4.0 International license (CC-BY 4.0).
