# Risk factor-based screening compared to universal screening for gestational diabetes mellitus in marginalized Burman and Karen populations on the Thailand-Myanmar border: An observational cohort

**DOI:** 10.12688/wellcomeopenres.17743.2

**Published:** 2023-01-18

**Authors:** Janna T. Prüst, Tobias Brummaier, Mu Wah, Htay Htay Yee, Nyo Nyo Win, Mupawjay Pimanpanarak, Aung Myat Min, Mary Ellen Gilder, Nay Win Tun, Onaedo Ilozumba, Basirudeen Syed Ahamed Kabeer, Annalisa Terranegra, Francois Nosten, Sue J. Lee, Rose McGready

**Affiliations:** 1 Shoklo Malaria Research Unit, Mahidol–Oxford Tropical Medicine Research Unit, Faculty of Tropical Medicine, Mahidol University, Bangkok, 10400, Thailand; 2 Department of Health Sciences, Vrije Universiteit Amsterdam, Amsterdam, 1081, The Netherlands; 3 Swiss Tropical and Public Health Institute, Allschwill, 4123, Switzerland; 4 University of Basel, Basel, 4001, Switzerland; 5 Department of Family Medicine, Faculty of Medicine, Chiang Mai University, Chiang Mai, 50200, Thailand; 6 Institute of Applied Health Research, College of Medical and Dental Sciences, University of Birmingham, Edgbaston, Birmingham, B15 2TT, UK; 7 Research Department, Sidra Medicine, Doha, Qatar; 8 Centre for Tropical Medicine and Global Health, Nuffield Department of Medicine, University of Oxford, Oxford, OX3 7LG, UK; 9 Mahidol-Oxford Tropical Medicine Research Unit, Mahidol University, Bangkok, 10400, Thailand

**Keywords:** Gestational diabetes mellitus, HAPO trial, Maternal and neonatal anthropometry, Oral glucose tolerance test, Symphysis-fundal height measurements, Migrants, Risk-factor-based screening, thin-diabetic

## Abstract

**Background:** Gestational diabetes mellitus (GDM) contributes to maternal and neonatal morbidity. As data from marginalized populations remains scarce, this study compares risk-factor-based to universal GDM screening in a low resource setting.

**Methods:** This is a secondary analysis of data from a prospective preterm birth cohort. Pregnant women were enrolled in the first trimester and completed a 75g oral glucose tolerance test (OGTT) at 24-32 weeks' gestation. To define GDM cases, Hyperglycaemia and Adverse Pregnancy Outcomes (HAPO trial) criteria were used. All GDM positive cases were treated. Sensitivity and specificity of risk-factor-based selection for screening (criteria: age ≥30y, obesity (Body mass index (BMI) ≥27.5kg/m ^2^), previous GDM, 1 ^st^ degree relative with diabetes, previous macrosomia (≥4kg), previous stillbirth, or symphysis-fundal height ≥90th percentile) was compared to universal screening using the OGTT as the gold standard. Adverse maternal and neonatal outcomes were compared by GDM status.

**Results:** GDM prevalence was 13.4% (50/374) (95% CI: 10.3-17.2). Three quarters of women had at least one risk factor (n=271 women), with 37/50 OGTT positive cases correctly identified: sensitivity 74.0% (59.7-85.4) and specificity 27.8% (3.0-33.0). Burman women (self-identified) accounted for 29.1% of the cohort population, but 38.0% of GDM cases. Percentiles for birthweight (p=0.004), head circumference (p=0.002), and weight-length ratio (p=0.030) were higher in newborns of GDM positive compared with non-GDM mothers. 21.7% (75/346) of newborns in the cohort were small-for-gestational age (≤10 ^th^ percentile). In Burman women, overweight/obese BMI was associated with a significantly increased adjusted odds ratio 5.03 (95% CI: 1.43-17.64) for GDM compared with normal weight, whereas in Karen women, the trend in association was similar but not significant (OR 2.36; 95% CI 0.95-5.89).

**Conclusions:** Risk-factor-based screening missed one in four GDM positive women. Considering the benefits of early detection of GDM and the limited additional cost of universal screening, a two-step screening program was implemented.

## Introduction

Gestational diabetes mellitus (GDM) is rising in tandem with obesity globally, including in South- and South-East Asia ^1^. The prevalence of GDM in Thailand is estimated between 6.1% and 29.2% ^1, 2^. In Myanmar, there is insufficient data to provide reliable estimations of the GDM prevalence ^1^. Detection of GDM is important as it is associated with neonatal macrosomia, neonatal hypoglycaemia and an increased risk for birth complications, such as shoulder dystocia and the need for caesarean section ^3– 5^. Furthermore, GDM is associated with an increased risk of preeclampsia, and entails a tenfold risk of developing type II diabetes and doubles the risk of cardiovascular events later in life ^6, 7^.

In absolute numbers more women are diagnosed with GDM in low- and middle-income countries (LMIC although relative estimates are similar between LMIC and high-income countries (HIC): 13.5% and 13.4%, respectively) ^8^. Within HIC, migrant women have a higher risk for GDM and associated adverse birth outcomes, but this is poorly evidenced for migrants in LMIC ^9^. In South-East Asia domestic as well as international migration is a dominant feature and access to health care for migrants is problematic ^10, 11^. While most women receive some form of antenatal care (ANC), screening for GDM is often not available ^12, 13^. In addition, awareness of GDM is limited, as are adequate protocols and tools to monitor blood glucose, which hinders best-practice management ^13, 14^.

Officially Thailand has approximately 2 million migrant workers predominantly from Myanmar, as well as an unknown number of undocumented migrants. Shoklo Malaria Research Unit (SMRU) has provided health care to both the refugee and migrant populations residing along the Thailand-Myanmar border. In the pregnant migrant population attending SMRU antenatal (ANC) clinics, the nutrition transition has been marked by a two-fold increase in first trimester overweight in just over a decade, aggravated by limited awareness of healthy diets and lifestyle ^15, 16^. These trends in marginalized populations are worrying given the greater risk of cardiometabolic effects occurring at lower BMI in Asians than in white Europeans ^17^.

In a meta-analysis, Lee *et al*. described a GDM prevalence of 11.5% in Asian women and identified the following risk factors: multiparity, previous GDM, or pregnancy-induced hypertension (PIH), a family history of GDM and an increased maternal body mass index (BMI ≥25kg/m ^2^) ^18^. An obstetric history of preterm birth, macrosomia, stillbirth, or an infant with congenital anomalies are also recognised GDM risk factors ^18^.

GDM diagnosis and management improves maternal and perinatal outcomes, although this is largely evidenced from HIC ^13, 19^. Both universal and risk-factor-based screening are common practices, with no international consensus about best practice ^2, 20, 21^. In 2011–2012, one of the first surveys conducted in a refugee camp reported a GDM prevalence of 10.1% (95% CI 6.2-14.0%) on the Thailand-Myanmar border with GDM being significantly associated with increased maternal age and parity, and low literacy ^20^. Although the proportion of caesarean section and obesity (BMI ≥27.5kg/m ^2^) were higher among women with GDM, this difference was not significant ^20^. In the low-resource setting of the refugee camp, the decision at that time was to commence efforts to screen for GDM based on risk factors using the Hyperglycaemia and Adverse Pregnancy Outcomes (HAPO) criteria ^22^. SMRU implemented this approach in all its antenatal care clinics on the border in 2018.

The study presented here aimed to evaluate the performance of two screening methods for GDM detection: risk-factor-based identification of pregnant women who were then screened by an OGTT, which was routinely used in antenatal care clinics for migrant women, to universal screening by OGTT. Within this cohort, risk factors for GDM were examined and adverse maternal and neonatal outcomes were evaluated in women with and without GDM.

## Methods

### Ethical approval

The study was approved by the ethics committee of the Faculty of Tropical Medicine, Mahidol University, Bangkok, Thailand (Ethics Reference: TMEC 15–062, initial approval 1 December 2015), the Oxford Tropical Research Ethics Committee (Ethics Reference: OxTREC: 33–15, initial approval 16 December 2015) and reviewed by the local Tak Province Community Ethics Advisory Board. The study was conducted in full conformity with the Declaration of Helsinki and followed regulations of the ICH Guidelines for Good Clinical Practice.

### Study design

This is a secondary analysis of data from an observational preterm birth cohort study with data collected prospectively between September 2016 and February 2019 in women enrolled in their first trimester of pregnancy (ClinicalTrials.gov Identifier: NCT02797327) with GDM screening occurring from December 2016 to November 2018.

### Study setting

SMRU was established more than three decades ago and combines research and humanitarian work that serves the migrant population alongside the Thailand-Myanmar border. To be accessible within these communities, which largely depend on below minimum wage jobs, SMRU operates free-of-charge walk-in clinics offering universal antenatal care, as well as 24-hour delivery services, led by trained personnel originating from the local population.

At the same clinics, women may be invited to participate in research. The study was explained to all pregnant women attending SMRU ANC clinics in the first trimester and they were invited to participate if they met the study inclusion criteria and enrolled if consent was forthcoming. Informed consent was obtained in the form of a signature or in the event of an illiterate participant by thumbprint coupled with a confirmatory signature by an impartial literate witness.

### Sample size

A detailed description of the study protocol and SMRU routine ANC procedures are available elsewhere ^23^. Briefly, women were followed fortnightly throughout pregnancy, at delivery, and in the postpartum period. The planned sample size of 400 in the original cohort study was based on estimated preterm birth rates (of approximately 8%) and on the following inclusion criteria: a viable, singleton first trimester pregnancy and an unremarkable medical and obstetric history e.g., no history of caesarean section. For this secondary analysis of the original cohort to determine appropriateness of GDM risk-factor-based screening, additional exclusion criteria were miscarriage prior to GDM screening, maternal death, lost to follow-up, withdrawal of consent (primary cohort), and if OGTT was performed late (gestational age (GA) ≥33 weeks) or not done at all. Women who did not complete follow-up to delivery were replaced as permitted in the original protocol. At an expected GDM rate of 10%, a sample size of 400 is expected to be sufficient to determine population prevalence ^20^.

### Study variables

Baseline characteristics, regular prenatal symphysis-fundal height (SFH) measurements, blood pressure, weight, and assessment of gestation by ultrasound, as well as birth outcomes, were collected by trained ANC staff and midwives in accordance with the study protocol. GA was estimated by crown rump length measured by first trimester ultrasound ^24^. Body-mass index (BMI) definitions followed recommendations for Asian BMI groups: underweight <18.5 kg/m ^2^; normal weight 18.5 to <23 kg/ m ^2^; overweight 23 to <27.5 kg/m ^2^; obese ≥27.5 kg/m ^2^ ^17^.

While the study protocol specified GDM screening with OGTT at 24–26 weeks of gestation, the HAPO study target time for testing was at 28 weeks (24–32 weeks) ^22^. Therefore, OGTTs to 32 weeks of gestation were included in this analysis. In women with a history of GDM, an OGTT was performed as early as possible in pregnancy and repeated at 24–26 weeks if previously negative. GDM diagnosis was based on HAPO trial cut-offs: a fasting capillary blood glucose measurement of ≥92mg/dL, ≥180mg/dL one hour or ≥153mg/dL two hours after ingestion of 75g glucose were considered positive ^22^.

### Risk-factor based screening

In 2018, risk-factor-based screening for GDM commenced at SMRU clinics. The risk factors were based on a survey in Karen and Burmese women in a SMRU refugee clinic screened at 24-28 weeks with a 75-gram OGTT using the HAPO trial cut-offs, where prevalence was 10.1% (95% CI 6.1-14.0). Risk factors in positive cases and review of recommendations from UK and Australia, both of which have populations of South-East Asian women, and Thailand resulted in the final list ^20^. The risk factors for GDM screening required at least one positive finding among the following 10 criteria: (i) age ≥30 years, (ii) obesity (BMI ≥27.5kg/m ^2^, the WHO definition for Asian populations) ^17^, (iii) GDM in a previous pregnancy, (iv) family history (1 ^st^ degree relative) of diabetes mellitus (although this is of reduced sensitivity in LMIC as access to diabetes screening is limited), (v) previous macrosomia (≥4kg), (vi) previous stillbirth, (vii) SFH ≥90th percentile, (viii) previous caesarean section regardless of birth-weight, (ix) 2+/3+ glucose on a urine dipstick test, or (x) polycystic ovarian syndrome (PCOS). The following criteria were not included in the analysis: women with a previous caesarean section, as they were excluded from the original study protocol, PCOS, as it was not encountered, and glucosuria, as there was no routine screening, leaving seven criteria.

### Maternal and Neonatal Outcomes

In resource-limited settings, assessment of the uterus size by SFH measurement as a proxy for fetal size has been suggested as a first level screening tool for fetal growth assessment. SFH measurement is a straightforward and inexpensive method, but its precision is controversial ^3^. A previously published bespoke SFH growth curve has been in use for more than 10 years in the pregnant population along the Thailand-Myanmar border ^25^; however, whether increased SFH using this local growth curve is a useful addition to the identification of GDM (macrosomia is a common adverse effect of GDM) has not been assessed.

Serial SFH measurements were included from 16 weeks of gestation on a two-weekly basis and data was examined using both, local population and international centiles ^25, 26^.

Gestational weight gain was defined as the final maternal weight measured not more than four weeks prior to birth, minus the weight measured at the first antenatal visit. For women with a normal BMI at enrolment (between 18.50 and 24.99kg/m ^2^), Intergrowth-21 ^st^ standard percentiles for each weight measurement from ≥26 weeks and ≤40 weeks of gestation were calculated ^27^.

Neonatal anthropometry (i.e., birthweight, head circumference, and length) were only considered if measured within 72 hours of birth. If women gave birth at SMRU, the neonate was weighed on a digital SECA 354 scale (precision 5g) with weekly calibration. Percentiles and z-scores for neonatal anthropometry were calculated using standards as published by the Intergrowth-21 ^st^ Project ^28^. Born too small or large for GA (SGA, LGA) were defined as ≤10 ^th^ and ≥90 ^th^ percentile, respectively.

Standard management of infants admitted to the special care baby unit included measurement of blood glucose and treatment for neonates with blood glucose below 45 mg/dL

### GDM management

If GDM was diagnosed, all women were counselled about lifestyle modification (e.g., diet and exercise) and, due to the unavailability of glucose self-monitoring in the population, the status of GDM control was monitored weekly or every two weeks at the clinic. Monitoring was as follows: women with GDM were asked to attend fasting and blood glucose was checked on arrival; then women ate a typical meal and were retested after one hour (post-prandial) with the desired value of <90 mg/dL (fasting) and <140 mg/dL (after one hour) for satisfactory control. Treatment was provided either directly or if non-pharmacologic interventions led to insufficient glucose control, with metformin as the first choice and glibenclamide as an additional oral agent. Due to the lack of home-based glucose monitoring options and the absence of adequate storage facilities, insulin is rarely prescribed in this population.

### Statistical analysis

Data were analysed using Stata, version 17.0 (TX, USA) (Stata, RRID:SCR_012763, https://www.stata.com/). Normally distributed continuous data were presented as means with standard deviation (SD) and non-normally distributed data as medians with interquartile range (IQR). Baseline characteristics as well as birth outcomes were compared between women with and without GDM. For continuous variables, the Student’s t-test or Mann-Whitney U test were used, and categorical variables were compared using the Fisher’s exact or Chi-square test. Univariate associations were quantified using logistic regression. To evaluate the predictive ability of the risk factors used in the current screening approach to identify women with GDM, all risk factors were combined into one logistic regression model, using GDM as the outcome. The sensitivity and specificity of risk-factor-based screening criteria was calculated using OGTT as the gold standard. An *any positive* test principle (i.e., if any of the GDM risk factors stated above were positive, an OGTT was performed) was the basis for this assessment. For further in-depth analysis and to identify risks and potential risk groups for GDM in this population, age (30 or older, vs. all others), smoking (yes/no), ethnicity (Karen and Burman), and BMI groups underweight, normal weight (reference group) and overweight/obese were explored using interaction terms and logistic regression modelling.

## Results

**Figure 1.**
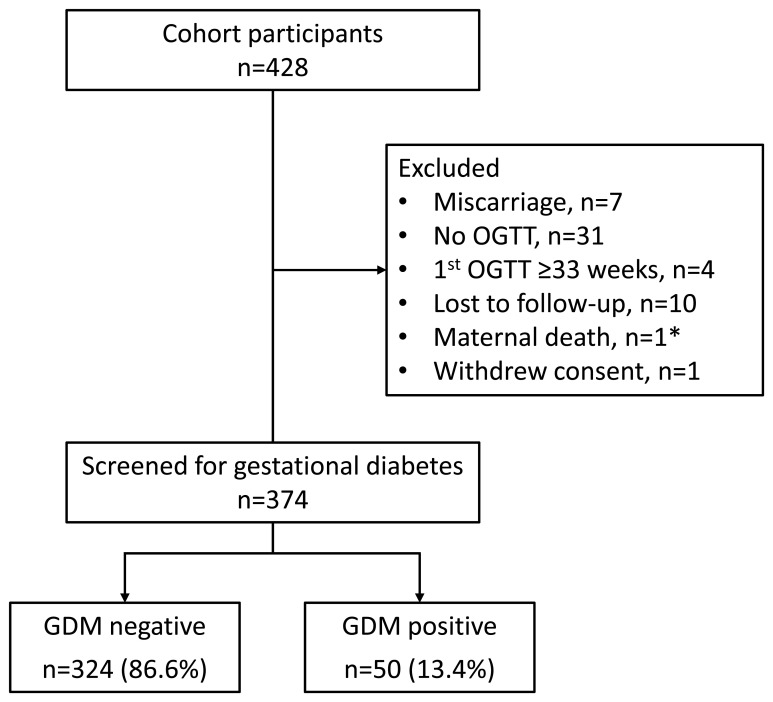
Flow diagram of participant selection. Abbreviations: GDM gestational diabetes mellitus, OGTT oral glucose tolerance test. * Sudden death due to mixed mitral valve disease at seven months gestation.

Following exclusions, 87.4% (374/428) of pregnant women from the original cohort were available for analysis ( Figure 1). Of these, 13.4% (50/374, 95% CI 10.3-17.2), were diagnosed with GDM by OGTT. The median number of antenatal care visits was 16 (IQR 15-17). Baseline maternal characteristics of women with and without GDM were compared ( Table 1). Women with GDM were significantly more likely to have had previous GDM (4.0% vs. 0, p<0.001) and postpartum hypertension (4.0% vs. 0.3%, p=0.006) and less likely to have had previous preterm labour (0% vs. 7.41%, p=0.047). A family history of diabetes was rarely reported (n=6) by women irrespective of GDM status.

**Table 1.** Demographic enrolment characteristics of women without and with GDM diagnosed by OGTT.

Characteristics	Total	Without GDM	With GDM	p-value
N	374	324	50	
Age (years), median [IQR]	25 [21, 30]	25 [21, 30]	24 [22, 28]	0.899
Age 30 and older, n (%) ^†^	99 (26.5%)	87 (26.9%)	12 (24.0%)	0.671
Ethnicity *, n (%)				0.333
Karen	247 (66.0%)	218 (67.3%)	29 (58.0%)	
Burman	109 (29.1%)	90 (27.8%)	19 (38.0%)	
Other	18 (4.8%)	16 (4.9%)	2 (4.0%)	
Gravidity, n (%)				0.935
Nulligravida	99 (26.5%)	86 (26.5%)	13 (26.0%)	
Multigravida	275 (73.5%)	238 (73.5%)	37 (74.0%)	
GA at enrolment (weeks), median [IQR]	9.6 [8.1, 11.6]	9.5 [8.0, 11.6]	9.9 [8.6, 11.7]	0.211
Literate, n (%)	240 (64.2%)	210 (64.8%)	30 (60.0%)	0.509
Smoking, n (%)	27 (7.2%)	21 (6.5%)	6 (12.0%)	0.161
BMI (kg/m ^2^), median [IQR]	20.6 [18.9, 23.3]	20.5 [19.0, 23.1]	21.0 [18.5, 24.4]	0.586
BMI ≥27.5kg/m ^2^, n (%) ^†^	23 (6.1%)	19 (5.9%)	4 (8.0%)	0.558
BMI <18.5kg/m ^2^, n (%)	73 (19.5%)	61 (18.8%)	12 (24.0%)	0.390
Height (cm), mean ± SD	151.8 ± 4.8	151.7 ± 4.8	152.4 ± 4.7	0.369
MUAC (cm), median [IQR]	25.9 [23.8, 28.3]	25.9 [23.9, 28.3]	25.4 [23.6, 28.9]	0.793
HIV, n (%)	0 (0.0%)	0 (0.0%)	0 (0.0%)	1.00
Syphilis, n (%)	6 (1.6%)	6 (1.9%)	0 (0.0%)	0.331
HepBsAg positive, n (%)	21 (5.6%)	17 (5.2%)	4 (8.0%)	0.431
Obstetric history, n (%)				
GDM ^†^	2 (0.5%)	0 (0.0%)	2 (4.0%)	<0.001
Vacuum delivery	3 (0.8%)	3 (0.9%)	0 (0.0%)	0.495
Macrosomia ^†^	2 (0.5%)	1 (0.3%)	1 (2.0%)	0.127
Stillbirth ^†^	6 (1.6%)	6 (1.9%)	0 (0.0%)	0.332
Miscarriage	93 (24.9%)	82 (25.3%)	11 (22.0%)	0.614
Previous preterm Labour	24 (6.4%)	24 (7.4%)	0 (0.0%)	0.047
Pregnancy Induced Hypertension	2 (0.5%)	2 (0.6%)	0 (0.0%)	0.577
Hypertension postpartum	3 (0.8%)	1 (0.3%)	2 (4.0%)	0.006
Family history of diabetes ^†^	6 (1.6%)	5 (1.5%)	1 (2.0%)	0.811
During Pregnancy				
SFH ≥90 ^th^ centile (GA ≥24), n (%) ** ^†^	205/374 (54.8%)	171/324 (52.8%)	34/50 (68.0%)	0.044
Gestational weight gain (kg), median [IQR]	10 [7, 12]	10 [7, 12]	10 [7, 12]	0.982
Weight gain ≥90 ^th^ centile	43/367 (11.7%)	38/319 (11.9%)	5/48 (10.4%)	0.764

Abbreviations (alphabetic order): Ag antigen, BMI body mass index, GA gestational age, GDM gestational diabetes mellitus, IQR interquartile range, HepBsAg hepatitis B surface antigen, HIV human immunodeficiency virus, MUAC mid-upper arm circumference, SD standard deviation.

† Included in list of risk-factor based screening

*Other includes Mon (n=8), Pa Oh (n=5), Rakhine (n=2), Shan (n=1), Ka Main (n=1), one patient self-identified as Muslim (n=1)

** at least once from 24 weeks onward

**Figure 2.**
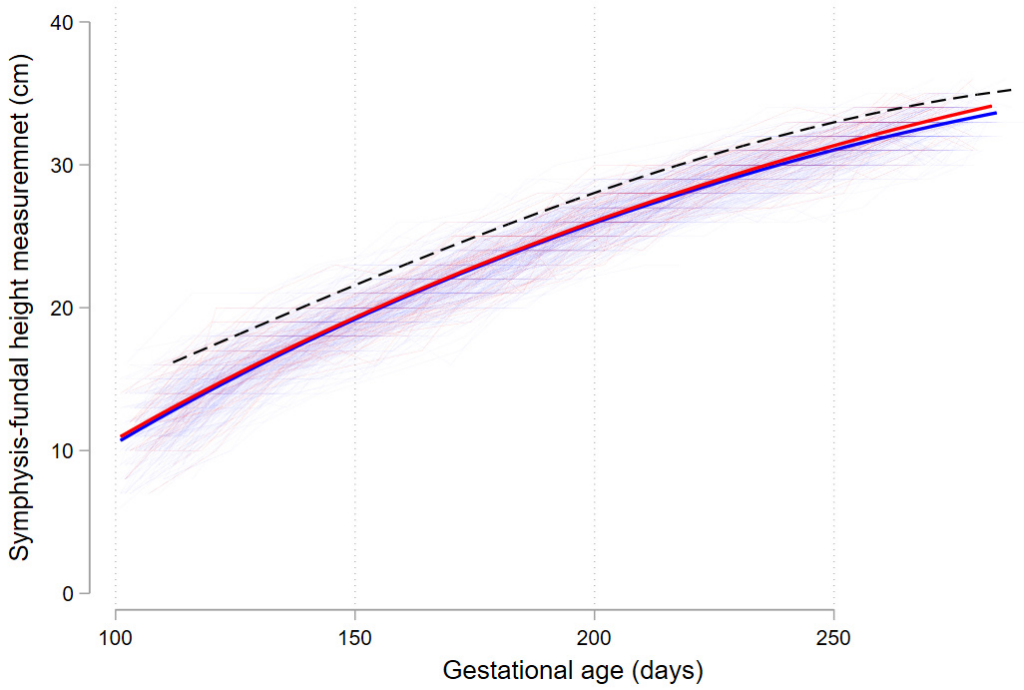
Symphysis-fundal height trajectories throughout pregnancy. Red lines indicate women with GDM (13.4%, n=50), blue lines women without GDM (86.6%, n=324). Dashed black line indicates the 90 ^th^ centile. Heavy red and blue lines represent fractional polynomial fit from individual measurements. Abbreviations: GDM gestational diabetes mellitus.

Overall, 23 women (6.1%) were obese (BMI ≥27.5kg/m ^2^). In the group of women who self-identified as being of Burman descent the GDM prevalence was 17.4% (19/109) compared to 11.7% (29/247) in women of Karen descent and 11.1% (2/18) in women of other ethnicities. Burman women accounted for 29.1% of the cohort population, but 38.0% of GDM cases ( Table 1). There were more women with GDM with an SFH ≥90 ^th^ centile during pregnancy with gestational week ≥24, 68.0% vs. 52.8%, p=0.044 ( Table 2). In particular, from about 224 days (32 weeks) onwards, women with GDM appeared to have larger SFH when compared with women without GDM ( Figure 2).

**Table 2.** Birth outcomes and neonatal anthropometry of women without and with GDM diagnosed by OGTT.

Birth outcomes and neonatal anthropometry	Total	Without GDM	With GDM	p-value
N	374	324	50	
GA at delivery (weeks), median [IQR]	39.6 [38.7, 40.1]	39.6 [38.8, 40.3]	39.1 [38.3, 39.9]	0.068
Gestational weight gain (kg), median [IQR]	10 [7, 12]	10 [7, 12]	10 [7, 12]	0.982
Weight gain ≥90 ^th^ centile	43/367 (11.7%)	38/319 (11.9%)	5/48 (10.4%)	0.764
SFH ≥90 ^th^ centile (GA ≥24), n (%)	205/374 (54.8%)	171/324 (52.8%)	34/50 (68.0%)	0.044
Preterm birth, n (%)	18/374 (4.8%)	17/324 (5.2%)	1/50 (2.0%)	0.318
Stillbirth, n (%)	4/374 (1.1%)	4/324 (1.2%)	0/50 (0.0%)	1.000
Mode of delivery				
Vaginal delivery, n (%)	352/374 (94.1%)	304/324 (93.8%)	48/50 (96.0%)	0.543
Caesarean Section, n (%)	20/374 (5.3%)	18/324 (5.6%)	2/50 (4.0%)	0.649
Place of labour				0.905
SMRU clinic, n (%)	301/374 (80.5%)	259/324 (79.9%)	42/50 (84.0%)	
Home, n (%)	27/374 (7.2%)	25/324 (7.7%)	2/50 (4.0%)	
Hospital, n (%)	37/374 (9.9%)	32/324 (9.9%)	5/50 (10.0%)	
Other, n (%)	9/374 (2.4%)	8/324 (2.5%)	1/50 (2.0%)	
Induction of labour, n (%)	25/373 (6.7%)	22/323 (6.8%)	3/50 (6.0%)	0.831
Augmentation of labour, n (%)	36/373 (9.7%)	31/323 (9.6%)	5/50 (10.0%)	0.929
Length of ROM (min), median [IQR]	36 [5, 160]	35 (5, 156)	65 (7, 217)	0.287
Postpartum haemorrhage ‡, n(%)	19/352 (5.4%)	18/304 (5.9%)	1/48 (2.1%)	0.274
Perineum				0.604
Intact, n (%)	160/303 (52.8%)	136/261 (52.1%)	24/42 (57.1%)	
1 ^st^ or 2 ^nd^ degree tear, n (%)	134/303 (44.2%)	116/261 (44.4%)	18/42 (42.9%)	
Episiotomy, n (%)	9/303 (3.0%)	9/261 (3.4%)	0/42 (0.0%)	
Infant sex (male), n (%)	181/373(48.5%)	155/323 (48.0%)	26/50 (52.0%)	0.597
Median Apgar score [IQR] at one min	9 [9, 9]	9 [9, 9]	9 [9, 9]	0.825
Median Apgar score [IQR] at five min	10 [10, 10]	10 [10, 10]	10 [10, 10]	0.620
Neonatal resuscitation, n (%)	8/361 (2.2%)	8/313 (2.6%)	0/48 (0.0%)	0.263
Abnormal newborn exam, n (%)	4/373 (1.1%)	4/323 (1.2%)	0/50 (0.0%)	1.00
Infant weight (g), mean ± SD	2972 ± 402	2952 ± 398	3096 ± 408	0.019
Large for GA (>p90), n (%)	7/346 (2.0%)	4/297 (1.3%)	3/49 (6.1%)	0.028
Small for GA (<P10), n (%)	75/346 (21.7%)	68/297 (22.9%)	7/49 (14.3%)	0.175
Percentile *, median [IQR]	24.8 [11.6, 47.6]	23.2 [11.2, 43.9]	40.5 [16.3, 61.0]	0.004
Head circumference, mean ± SD	32.8 ± 1.3	32.7 ± 1.3	33.3 ± 1.3	0.005
Percentile, median [IQR]	19.9 [7.54, 40.4] ^†^	19.3 [6.69, 37.6] ^§^	30.6 [12.8, 60.5] ^$^	0.002
Length ^28^, mean ± SD	48.2 ± 2.0	48.1 ± 2.0	48.4 ± 1.8	0.358
Percentile, median [IQR]	27.5 [13.2, 50.4] ^†^	26.5 [13.0, 49.3] ^§^	33.1 [15.6, 59.8] ^$^	0.182
Weight-length ratio (%), mean ± SD	6.2 ± 0.7	6.1 ± 0.7	6.4 ± 0.7	0.010
Percentile, median [IQR]	0.96 [0.61, 1.72] ^†^	0.91 [0.60, 1.62] ^§^	1.17 [0.91, 1.92] ^$^	0.030
Admitted special care baby unit, n (%)	73/374 (19.5%)	65 (20.1%)	8 (16.0%)	0.500
Hypoglycaemic, n (%)	2/73 (2.74%)	2/65 (3.08%)	0/8	0.615

Abbreviations (alphabetic order): GA gestational age, GDM gestational diabetes mellitus, IQR interquartile range, min minutes, ROM rupture of membranes, SD standard deviation, SFH symphysis fundal height, SMRU Shoklo Malaria Research Unit.

*birth weight for GA and sex, ‡ >500ml blood loss, † n=345, § n=296, $ n=49

### Birth outcomes

Newborns from mothers with GDM were heavier (mean birthweight (SD): 3096g (408) vs. 2952g (398), p=0.019), and nearly five times more likely to be born large for gestational age (6.1% (3/49) vs. 1.3% (4/297), OR 4.78, 95% CI 1.04-22.1) ( Table 2). They were also more likely to be in a higher percentile for birthweight and head circumference, adjusted for GA and sex: median [IQR]: 40.5 [16.3, 61.0] vs. 23.2 [11.2, 43.9], p=0.004), and 30.6 [12.8, 60.5] vs. 19.3 [6.69, 37.6], ^, ^p=0.002 respectively. Infants born to mothers with GDM had a higher weight-length ratio (mean (SD): 6.4% WLR (0.7) vs. 6.1% w/l (0.7), p=0.010), Table 2. Overall, the proportion of SGA was relatively high (21.7%, 75/346) with a lower proportion of SGA in the GDM positive group which was not statistically significant (14.3% (7/49) vs. 22.9% (68/297, p=0.175). Other adverse birth complications such as stillbirth (0%, 0/50 of GDM positive; 1.2%, 4/324 of GDM negative), and preterm birth (2.0%, 1/50 in GDM positive; 5.2%, 17/324 in GDM negative) were low.

### OGTT test results

As expected, the absolute blood sugar levels (BSL) levels were higher in the GDM positive group ( Table 3). Of the women with GDM, 88.0% (44/50) had only one of the three glucose measurements above the cut-off, 10% (5/50) had two of three glucose measurements above the defined threshold and in only one study participant (1/50, 2.0%) all three measurements were above the defined limits. Screening with fasting and two-hour results, as performed in some institutions to reduce costs, would result in only 66% (33/50) of the GDM cases being detected in this study population ( Table 3).

**Table 3.** Details of OGTT test result and GDM treatment.

OGTT test results and GDM treatment	Total	Without GDM	With GDM	p-value
N	374	324	50	
GA (weeks) at OGTT, median [IQR]	26.6 [25.7, 27.6]	26.6 [25.7, 27.6]	26.6 [25.9, 27.4]	0.949
OGTT * results (mg/dL), median [IQR]				
BSL fasting	79 [74, 84]	78 [73, 83]	86 [81, 96]	<0.001
BSL one hour	132 [114, 154]	129 [112, 147]	173 [142, 191]	<0.001
BSL two hours	111 [97, 127]	110 [96, 123]	129 [113, 157]	<0.001
Proportion of positivity at each OGTT timepoint				
Fasting only			17 (34%)	
One hour only			17 (34%)	
Two hours only			10 (20%)	
Fasting and one hour			2 (4%)	
Fasting and two hours			0 (0%)	
One hour and two hours			3 (6%)	
All three			1 (2%)	
GDM treatment, n (%)				
Diet and exercise only			18 (36%)	
Diet & metformin			27 (54%)	
Metformin and glibenclamide			4 (8%)	
Metformin and insulin			1 (2%)	

Abbreviations (alphabetic order): BSL blood sugar level, GA gestational age, GDM gestational diabetes mellitus, HAPO Hyperglycaemia and Adverse Pregnancy Outcomes, IQR interquartile range, OGTT oral glucose tolerance test.

*HAPO cut points in GDM: fasting, one hour and two hours BSL are ≥92, ≥180 and ≥153mg/dL, respectively.

### Risk-factor-based screening for GDM

There were 37 women in the GDM positive group and 234 women in the GDM negative group who had at least one risk factor, translating into an overall proportion of 72.5% (271/374) ( Table 1). Of the 50 OGTT positive cases, 37 were correctly identified by risk factors alone, resulting in a sensitivity of 74.0% (59.7%-85.4%). Specificity was low, with 90 of 324 being correctly identified as negative for GDM using risk-factor-based screening: 27.8% (23.0%-33.0%). The positive and negative predictive values were 13.7% (9.8%-18.3%) and 87.4% (79.4%-93.1%), respectively.

Of the seven risk-factor-based screening items included in this analysis, a history of GDM and previous stillbirth could not be included in a multivariable model due to zero counts. None of the risk-factor-based screening criteria significantly predicted GDM status in this migrant population. History of macrosomia had a positive (wide confidence interval) and non-significant association due to the small number of cases (6.59, 95% CI 0.41-107.1, p=0.185). All other risk factors were not significant at p>0.20.

### GDM management and treatment

Approximately two out of three women, 64% (32/50), were medicated for their GDM ( Table 3). Most received metformin only (54% (27/50)), with a smaller proportion receiving metformin plus glibenclamide (8.0% (4/50)), and only one patient (2.0%) received insulin due to metformin failure at 27+3 weeks of gestation. This case required referral to the government hospital.

### GDM risk in Burman and Karen ethnic groups

Risk factors for GDM were examined separately for the two main ethnic groups in the population by multivariate analysis ( Table 4). After adjustment, overweight or obese Burman women were at a five-fold higher risk of GDM. A different relationship between BMI and GDM was apparent for Karen women where the risks were similarly elevated (non-significant) for both underweight and overweight or obese women ( Table 4).

**Table 4.** Risk factors for GDM diagnosed by OGTT in Karen and Burman women.

Risk factors	Karen n=247				Burman n=109			
	No GDM, n=218	GDM, n=29	Adjusted Odd Ratio (95% CI)	p-value	No GDM, n=90	GDM, n=19	Adjusted Odd Ratio (95% CI)	P-value
Age 30 and older, n (%)	56 (25.7)	6 (20.7)	0.52 (0.18-1.52)	0.231	24 (26.7)	5 (26.3)	0.54 (0.15-1.92)	0.343
Smoker, n (%)	19 (8.72)	5 (17.2)	3.09 (0.92-10.39)	0.069	2 (2.22)	1 (5.26)	5.27 (0.39-71.88)	0.213
BMI, kg/m ^2^ *								
Normal (18.50-22.99)	126 (57.8)	11 (38.0)	reference		46 (51.1)	6 (31.6)	reference	
Underweight (≤18.5)	31 (14.2)	7 (24.1)	2.41 (0.85-6.79)	0.097	26 (28.9)	4 (21.1)	1.20 (0.30-4.73)	0.704
Overweight / obese (≥23)	61 (28.0)	11 (37.9)	2.36 (0.95-5.89)	0.064	18 (20.0)	9 (47.4)	**5.03 (1.43-17.64)**	**0.012**

Data are shown in n (%) unless otherwise indicated. Abbreviations (alphabetic order): BMI body mass index, GDM gestational diabetes mellitus.

* BMI definitions followed recommendations for Asian BMI groups.

## Discussion

The most consequential early GDM definition was published by O´Sullivan and Mahan in 1964 ^29^. Their criteria were then tried and adapted over decades with the culmination in the HAPO trial ^30, 31^. Currently there is no consensus on the optimal screening approach with Europe leaning more to risk-factor based screening and USA towards glucose challenge tests; and it is not entirely clear whether criteria derived from high-resource settings are adequate for institutions in low-resource settings ^30, 32^. Hence, as the main objective of this manuscript was to assess the performance of the risk-factor-based screening used in routine clinical practice and draw conclusions of its fitness, the presented cohort was explored by an *any positive* approach. This was possible because all women had data on the relevant risk-factors collected, and as they were part of a preterm birth study cohort, all women had an OGTT done. The analysis identified the shortcomings of current clinical practice as almost one in four women with GDM would have been missed based on risk-factor-based selection for screening when compared with universal screening by 75g OGTT.

While the risk-factor-based screening had a sensitivity of 74.0% (95% CI 59.7-85.4), it lacked specificity 27.8% (95% CI 23.0-33.0) and resulted in an inadequate positive predictive value of 13.7% (95% CI 9.8-18.3). Reasons for this underperformance could be related to the limited size of the cohort; due to exclusion of women with a previous caesarean section (potentially due to undiagnosed GDM) from the original cohort; or that risk-factor-based screening is inherently weak for GDM diagnosis in South-East Asian women. The low incidence of reported prior history of GDM or family history of diabetes, most likely results from the limited extent of testing in this population that has limited access to health care ^33^.

At least one in seven ‘healthy’ migrant women presenting to antenatal care in this study cohort had GDM based on the 75g OGTT and thus identifying GDM as a significant health problem in Burman and Karen migrants on the Thailand-Myanmar border. These findings are similar to other migrant populations globally who have to make food choices based on limited expenditure ^34^. The BMI-related differences in risk factors observed on regression analysis for GDM in Karen and Burman women may relate to different diets and smoking habits between these ethnic groups. A more detailed dietary analysis based on quantitative 24-hour food recall is currently under evaluation. The similar odds for GDM in underweight and overweight/obese Karen women may be related to the thin-type II diabetic phenotype where individuals are at increased risk at a lower BMI ^35^. Gujral *et al*. and Rajakramikan *et al.* have proposed pathogenic mechanisms including impaired insulin secretion, *in utero* undernutrition, or epigenetic alterations, to explain thin-type II diabetes ^36, 37^. Of greatest concern is the propensity for this group of patients with undernutrition to have worse diabetes. Ethnohistorical Burman and Karen are distinct populations with their own pheno- and genotypic peculiarities ^38^. As the slightly different GDM risk-profile is based on a small sample size, these findings must be confirmed in larger cohorts.

In this analysis, there was a positive association between GDM and higher percentiles for infant birthweight, larger head circumference and weight-length ratio composition but no difference was seen in mode of delivery, postpartum haemorrhage, perineal damage or Apgar score by GDM status ^39– 41^. Given that pregnant women with an unremarkable medical and obstetric history were prioritized in the cohort and women with GDM received treatment following the abnormal OGTT result, the low rate of adverse birth outcomes is not unexpected. The high rate of small for gestational age (one in five) newborns has been reported previously and highlights the double burden of nutrition in this population but may also signal a risk for thin-type II diabetes ^15, 35^. Data from other South-East Asian populations suggest that obese women with GDM have a higher risk of adverse outcome when compared to normal weight pregnant women with GDM ^42^. However, considering a significant increase in perinatal morbidity in women with uncontrolled GDM compared to women with adequately treated GDM, different strategies of GDM management for obese and non-obese pregnant women does not seem appropriate at this point ^43^.

Early detection of GDM may prevent the need for caesarean section, which limits total expenditure per pregnancy. While the cost for an individual OGTT is small (i.e., approximately 18 THB (0.54 USD) for one glucose test strip, 7.5 THB (0.22 USD) for 75g glucose powder), costs add up if thousands of pregnant women are universally screened each year. Considering the average cost for caesarean section in 2020 for migrant women was 27,695 THB (approximately 824 USD) when referred to the public hospital system, one averted caesarean section would be equivalent to 1,539 glucose test strips – enough for OGTTs in 500 women. Mo *et al*. concluded that cost effectiveness of universal GDM screening is likely favourable over screening of targeted high-risk populations in a meta-analysis in mostly HIC, while others suggest that universal screening is not useful ^44, 45^. Since access to adequate diabetes monitoring and pharmacological intervention is severely limited outside of pregnancy in resource-limited settings, there may be added benefit to universal screening in LMIC. The counselling women receive during pregnancy about their GDM may be the first and only information provided on lifestyle modification to prevent the development of type II diabetes later in life ^46^. Reducing from three (fasting, one hour, two hours) to two (fasting, two hours) tests to bring down costs is not a useful alternative in this population as nearly nine in 10 were positive at a single timepoint distributed across all three time points. As the majority (68.7%) of GDM positive women in this study used oral hypoglycaemic agents, there is a need for a better understanding of effective lifestyle interventions in this marginalized group ^2, 16, 47^.

The findings on the usefulness of SFH contributes to the ongoing debate on the use of international vs. local centiles. The proportion of pregnant women presenting with a SFH ≥90 ^th^ centile using local centiles differs markedly compared to the proportion when using international centiles. Using international standards for SFH, most GDM positive women would not be signalled as women with a problem in this population ^26^. This most likely arises from maternal anthropometric differences (e.g., the greater than 10cm difference in maternal height) between the populations participating to the cohorts for centile curve calculation.

From 24 weeks EGA there was a significantly higher proportion of women in the GDM positive group with a SFH above the 90 ^th^ centile compared to women without GDM. While this suggests that SFH may have a role, the fact that more than half (52,8%) the women with no GDM had at least one SFH measurement ≥90 ^th^ centile renders SFH for GDM as rather unspecific. In addition, the timeframe of detection of increased SFH (32 weeks) is later than when an OGTT identifies GDM.

### Strengths of this study

The strengths of this study include first trimester enrolment and ultrasound dating allowing accurate assessment of neonatal anthropometry based on gestation. The risk of information bias is reduced by the prospective cohort design with minimal missing data. There was also close monitoring throughout pregnancy with a high number of antenatal care visits (median 16, IQR 15-17). Furthermore, weight and SFH were measured with calibrated instruments and by well-trained personnel. In addition, this analysis has had a direct local impact resulting in the implementation of universal GDM screening for all women with a two-step approach; with the first step being a glucose challenge test (i.e., 50 g non-fasting oral glucose load, followed by a 1-hour glucose measurement) with 1-hour levels of ≥200 mg/dL being diagnostic of GDM and values 140–199 mg/dL requiring a 2 ^nd ^step, namely a complete OGTT. This pragmatic choice to increase the number of women screened and minimize the burden of a full OGTT in all women follows the recommendation of the American College of Obstetricians and Gynecologists.

### Potential study limitations

Women with a complicated obstetric or medical history were excluded from the original study. As SMRU does not perform caesarean sections in their clinics, women thought to be at risk of this pregnancy complication were excluded from the original study as they were predicted to not be able to provide a complete set of samples. This was a selection bias for healthier pregnant women, potentially leading to an underestimate of the GDM prevalence in this border population, i.e., the study likely presents the minimum GDM rate in the community of pregnant women. With the selection bias and treatment for all GDM positive women there was a low number of complications; among 37 risk-factor positive cases (vs 17 risk-factor negative cases), there were seven complications overall (preterm (n=1), stillbirths (n=0), caesarean section (n=2), postpartum haemorrhage (n=1), and LGA (n=3)). The study design did not allow exploration of whether those identified in the high-risk group were also the same women who are likely to have complications from GDM. 

Due to the relatively small sample, the suggestion of differences in the risk of GDM between the two major ethnic groups requires further verification.

## Conclusions

These findings imply that GDM is a problem at the Thailand-Myanmar border with Burman women who are overweight/obese being at the highest risk. GDM determined by risk-factor-based screening performed sub-optimally in this rural, resource-constrained pregnant population. Access to universal screening for GDM can potentially reduce negative impacts for an individual pregnancy but also provide an opportunity to sensitize people in marginalized populations of their potential increased risk for type II diabetes later in life. Considering that additional costs for universal screening appear limited, this is the preferred policy in this population.

## Data Availability

Oxford University Research Archives: MSP COHORT GDM SCREEN. Data are available under the terms of the Creative Commons Attribution 4.0 International license (CC-BY 4.0). **Figshare: STARD checklist for** ‘ Risk factor-based screening compared to universal screening for gestational diabetes mellitus in marginalized Burman and Karen populations on the Thailand-Myanmar border: an observational cohort’. https://doi.org/10.6084/m9.figshare.19382624 ^48^.
