# Supplementary material for: Risk factor-based screening compared to universal screening for gestational diabetes mellitus in marginalized Burman and Karen populations on the Thailand-Myanmar border: An observational cohort
Source: Wellcome Open Res. 2023 Jan 18;7:132. Originally published 2022 Apr 7. [Version 2] doi: 10.12688/wellcomeopenres.17743.2 (PMC9976631; doi:10.12688/wellcomeopenres.17743.2)
Supplement: Peer review history

# Peer review: Reviewer response for version 2

**DOI:** 10.21956/wellcomeopenres.20890.r54240

**Published:** 2023-02-22

**Authors:** Blair Johnson Wylie

**Affiliations:** 1 Department of Obstetrics and Gynecology, Beth Israel Deaconess Medical Center, Boston, MA, USA

These changes are acceptable to me. I approve the revisions

Is the work clearly and accurately presented and does it cite the current literature?

Partly

If applicable, is the statistical analysis and its interpretation appropriate?

Partly

Are all the source data underlying the results available to ensure full reproducibility?

Yes

Is the study design appropriate and is the work technically sound?

Partly

Are the conclusions drawn adequately supported by the results?

Partly

Are sufficient details of methods and analysis provided to allow replication by others?

Partly

Reviewer Expertise:

NA

I confirm that I have read this submission and believe that I have an appropriate level of expertise to confirm that it is of an acceptable scientific standard.

# Peer review: Reviewer response for version 2

**DOI:** 10.21956/wellcomeopenres.20890.r54239

**Published:** 2023-02-21

**Authors:** Jane E. Hirst

**Affiliations:** 1 Nuffield Department of Women's & Reproductive Health, University of Oxford, Oxford, UK

Thank you for the opportunity to look at this revision. The paper addresses a topic that has not previously been well described in this population. It is well written and presented and I have no further comments for the authors and recommend indexing.

Is the work clearly and accurately presented and does it cite the current literature?

Partly

If applicable, is the statistical analysis and its interpretation appropriate?

Partly

Are all the source data underlying the results available to ensure full reproducibility?

Yes

Is the study design appropriate and is the work technically sound?

Yes

Are the conclusions drawn adequately supported by the results?

No

Are sufficient details of methods and analysis provided to allow replication by others?

Partly

Reviewer Expertise:

NA

I confirm that I have read this submission and believe that I have an appropriate level of expertise to confirm that it is of an acceptable scientific standard.

# Peer review: Reviewer response for version 1

**DOI:** 10.21956/wellcomeopenres.19636.r52286

**Published:** 2022-09-23

**Authors:** Blair Johnson Wylie

**Affiliations:** 1 Department of Obstetrics and Gynecology, Beth Israel Deaconess Medical Center, Boston, MA, USA

The authors should be commended for tackling the issue of GDM in a vulnerable and marginalized population of migrants/refugees on the Thai-Myanmar border. Much of the evidence underlying practice recommendations for GDM comes from high-resource populations. The title suggests the analysis will focus on the comparison of risk factors vs universal GDM screening; this was a question posed in HIC in the not-too-distant past with evidence/practice moving to universal screening. Addressing this question in this population is novel. However, the data presented cover a number of GDM-related topics. As presented though, it is a bit challenging for the reader to pull out the key questions and conclusions.

 From my reading of the manuscript, there are a number of questions being posed:

- Prevalence of GDM (with universal screening)
- What are the risk factors for GDM in this population
- Comparison of risk factor-based approach (risk factors identified prior to this study) with universal screening (test performance characteristics)
- Association of GDM with adverse pregnancy outcomes
- Seasonality of GDM diagnoses
- Serial SFH and GDM

I think the overarching scientific question is whether in this population GDM should be evaluated and screened for like it is in high-income settings. Data may not be sufficient to answer this.

 Main criticism/suggestion - clarify the specific question(s) being posed in the manuscript. Some of the topics may need to be saved (and extended) in separate manuscripts.  In particular, the seasonality of GDM diagnosis, while intriguing, seems misplaced and not fully developed. The serial SFH analysis also seems perhaps unnecessary (at least in the figure).

 Specific suggestions:

1. Is this a secondary analysis of a prospective cohort constructed for another purpose (this was unclear in the abstract and methods)?
2. Remove “etc” from the abstract.
3. Clarify if this was a “homegrown” risk factor-based approach or not.
4. Why was obesity defined as BMI greater than 27.5?
5. Better clarification is needed about the analysis of risk factor exploration for GDM and how this is distinct from the risk factor-based screening. 
6. The first sentence of the second paragraph is confusing as written; the prevalence same, but more people living in LMICs so the overall number is higher.
7. The introduction is a bit long and may not need to be. The connection with malnutrition in the introduction is not entirely clear. The paragraph on SFH seems extraneous. The paragraph on GDM and the environment is interesting but a little off-topic in the manuscript.
8. Intro sentence that states “adequate diagnosis and mgmt. improves outcomes in GDM”—is this known for LMICs or migrant populations? It might not improve outcomes—this article helps contributes to that literature but is not sufficient to answer the question.
9. Methods section detailing screening using HAPO criteria— sixth paragraph— seems to reference #24 twice in the same sentence.
10. Sample size calculations unclear—likely as unclear as written the purpose of the primary cohort.
11. Exclusion criteria—one is listed as miscarriage. Is this history of miscarriage or pregnancy loss before glucose screening in this population?
12. Methods—could eliminate some of the details of SFH measurements and reference prior work by this group.
13. Details of newborn anthropometry measurements—some could be relegated to a supplement to simplify the manuscript.
14. How define LGA? Based on INTERGROWTH?
15. Details of analytic plan and modeling insufficient—why is a multivariable model needed? As comparing the RF-based approach (1 RF buys you screening) rather than creating a prediction model to model the probability of GDM diagnosis. The model may be overfitted.
16. Why interaction terms? What effect modification is being explored?
17. P-values to 3 decimal places probably can be taken to only 1 or 2 places based on journal guidelines.
18. Consider eliminating Figure 2.
19. The % of LGA is remarkably small in this population—this is a worth finding worth highlighting. Are the risks of GDM therefore the same in a population with less LGA and less maternal overweight?
20. Consider adding some historical context of literature from HIC and the switch from RF-based screening to universal.
21. Consider adding some historical context of the debate around whether to screen for GDM at all—expensive, onerous, does it meaningfully improve outcomes?  Have we yet answered this for LMICs?
22. What proportion of women have at least one risk factor— would it be almost universal?
23. The paragraph on SFH in conclusions is a bit hard to understand.

Is the work clearly and accurately presented and does it cite the current literature?

Partly

If applicable, is the statistical analysis and its interpretation appropriate?

Partly

Are all the source data underlying the results available to ensure full reproducibility?

Yes

Is the study design appropriate and is the work technically sound?

Partly

Are the conclusions drawn adequately supported by the results?

Partly

Are sufficient details of methods and analysis provided to allow replication by others?

Partly

Reviewer Expertise:

NA

I confirm that I have read this submission and believe that I have an appropriate level of expertise to confirm that it is of an acceptable scientific standard, however I have significant reservations, as outlined above.

# Peer review: Reviewer response for version 1

**DOI:** 10.21956/wellcomeopenres.19636.r51816

**Published:** 2022-08-10

**Authors:** Jane E. Hirst

**Affiliations:** 1 Nuffield Department of Women's & Reproductive Health, University of Oxford, Oxford, UK

Thank you for this interesting article. I have raised a few points below to clarify the objective of the paper and the interpretation. Overall, the study would be stronger if it were framed as an exploratory study to understand the clinical picture and patterns of GDM in this population. This would make the seasonality data more obviously relevant.

 Points to address:

**Abstract:**

1. In the abstract, it states "From the prospective cohort...", this is confusing as it implies another study. It would be clearer to state the study design was an observational cohort study to study preterm birth and this was a secondary analysis. It would be helpful to define "Healthy" for this study: does this mean women without prior or current medical complications?
2. Please list the 10 risk factors as it is unclear with just the top most well-known RF what the others are.
3. It seems strange to report the non-significant association between GDM and underweight and overweight/obese Karen women. I question whether this is helpful in the results.
4. The conclusion states that risk factors screening was not sufficiently sensitive or specific, however, these rates are similar to what is used in many higher-income countries, including the UK. The question should be whether those identified in the high-risk group are also the same women who are likely to have complications from GDM, thus warranting treatment.

**Introduction**

1. The introduction is quite long, making the narrative of the paper difficult to discern for a busy reader. The section on the environment, for example, it is unclear how it directly relates to what you present here.

**Methods**

1. Sample size: Whilst you give a rationale for the wider cohort study sample and describe pragmatically within this sample how many women were included, you do not give any indication as to whether this study was adequately powered to determine population prevalence or not.
2. Here you state that only seven criteria were used for risk factor screening. This should be corrected in the abstract.
3. Did you collect variables on any other complications associated with GDM other than the newborn size at birth, e.g. neonatal hypoglycaemia, primary CS, stillbirth? I note that BMI is defined in the results using Asian centiles. This should be specified in the methods.
4. GDM management: Was the weekly or fortnightly monitoring of glucose fasting post-prandial or random?

**Results**

1. You report non-significant differences in baseline characteristics, which is confusing to the reader. It would be clearer to state there were no significant differences observed in those variables, or alternately that a null association cannot be excluded.
2. Again, be careful reporting non-significant trends in the difference in prevalence between ethnic groups.
3. Figure 2: The lines seem pretty much the same to me. Is this a significant difference? It would be useful to articulate this in cm difference if it is clinically relevant.
4. When reporting the birth outcomes, it is important to know gestational age at delivery as the difference in birth weight may be gestation related. 
5. The CI for LGA is very large indicating not many babies were LGA. It would be helpful for the reader to have the absolute number of non-GDM and GDM LGA babies in the text. The fact that so many more GDM babies are SGA should also be highlighted here.
6. In the risk factors-based screening for GDM section, you start by reporting the sensitivity of the different tests in the OGTT. This should have been pre-empted in the methods and would be better under a subheading about OGTT (relating to Table 3).
7. I am a bit confused as you report risk factor screening has 74% sensitivity and 27.8% specificity, however, in the results, you then state none of the risk factors was associated with the outcome (GDM). Does this mean that women with GDM had more than one risk factor and this was the difference? What was your definition of a risk factor for the calculation of sensitivity and specificity? If you are using the OGTT values themselves, doesn't that defeat the purpose of risk factor-based screening?
8. Table 4: I worry that you are splitting your sample size and with multiple testing, it is not surprising that eventually one of your tests came up positive. There is no discussion of how you will handle false detection rates in the methods. 
9. The information on seasonality is interesting, although it is unclear how it relates to the study objective. Did you include seasonality as a risk factor?

**Discussion**

1. I disagree that the risk factors-based screening was "grossly inadequate", although as per my comments above I am confused as to what the risk factors were that were included in your screening tests. You did not show any great changes in perinatal outcomes, other than a slight increase in the birthweight of babies, which in a setting with such high rates of LGA may not be a bad thing in itself.
2. The value of screening and treating GDM in non-obese Asian populations has been questioned (see Yue *et al.,* BMC Pregnancy and Childbirth, 2022 ^1^). Whilst I appreciate that an everted CS will save a lot of money, the question that arises from this is with such low CS rates (around 5% overall), how much can you extrapolate from data from HAPO derived in very differently resource settings?

Is the work clearly and accurately presented and does it cite the current literature?

Partly

If applicable, is the statistical analysis and its interpretation appropriate?

Partly

Are all the source data underlying the results available to ensure full reproducibility?

Yes

Is the study design appropriate and is the work technically sound?

Yes

Are the conclusions drawn adequately supported by the results?

No

Are sufficient details of methods and analysis provided to allow replication by others?

Partly

Reviewer Expertise:

Obstetrics, diabetes in pregnancy, global health

I confirm that I have read this submission and believe that I have an appropriate level of expertise to confirm that it is of an acceptable scientific standard, however I have significant reservations, as outlined above.

## References

[1] : Clinical consequences of gestational diabetes mellitus and maternal obesity as defined by asian BMI thresholds in Viet Nam: a prospective, hospital-based, cohort study. *BMC Pregnancy Childbirth* .2022;22(1) : 10.1186/s12884-022-04533-1 195 10.1186/s12884-022-04533-1 35264148PMC8908699
